# Clinical application of advances and innovation in radiation treatment of hepatocellular carcinoma

**Published:** 2021-11-06

**Authors:** Valerie J. W. Tong, Vishal G. Shelat, Yew Kuo Chao

**Affiliations:** ^1^Yong Loo Lin School of Medicine, National University of Singapore, Singapore; ^2^Department of General Surgery, Tan Tock Seng Hospital, 308433, Singapore; ^3^Department of Gastroenterology and Hepatology, Tan Tock Seng Hospital, 308433, Singapore

**Keywords:** hepatocellular carcinoma, radiation therapy, hepatology, general surgery, oncology

## Abstract

**Background:**

Hepatocellular carcinoma (HCC) management has evolved over the past two decades, with the development of newer treatment modalities. While various options are available, unmet needs are reflected through the mixed treatment outcome for intermediate-stage HCC. As HCC is radiosensitive, radiation therapies have a significant role in management. Radiation therapies offer local control for unresectable lesions and for patients who are not surgical candidates. Radiotherapy also provides palliation in metastatic disease, and acts as a bridge to resection and transplantation in selected patients. Advancements in radiotherapy modalities offer improved dose planning and targeted delivery, allowing for better tumor response and safer dose escalations while minimizing the risks of radiation-induced liver damage. Radiotherapy modalities are broadly classified into external beam radiation therapy and selective internal radiation therapy. With emerging modalities, radiotherapy plays a complementary role in the multidisciplinary care of HCC patients.

**Aim:**

We aim to provide an overview of the role and clinical application of radiation therapies in HCC management.

**Relevance for Patients:**

The continuous evolution of radiotherapy techniques allows for improved therapeutic outcomes while mitigating unwanted adverse effects, making it an attractive modality in HCC management. Rigorous clinical studies, quality research and comprehensive datasets will further its application in the present era of evidence-based practice in Medicine.

## 1. Introduction

Hepatocellular carcinoma (HCC) is one of the top five human cancers and an important public health problem. HCC management is evolving over the past two decades. The Barcelona-Clinic Liver Cancer (BCLC) staging system is the most commonly used classification to guide HCC management and considers three prognostic variables: Tumor stage, presence of cancer-related symptoms, and degree of liver dysfunction, and predicts treatment outcome [1]. The tumor stage is assessed by imaging, and cancer-related symptoms are determined by the Eastern Cooperative Oncology Group performance status. Liver function was previously represented by Child-Pugh score (or Child’s score), but portal venous pressure is added in the 2018 update [1]. The BCLC staging system is endorsed by both the American Association for the Study of Liver Diseases (AASLD) and the European Association for the Study of the Liver (EASL) [2,3]. Patients are grouped into very early-(BCLC 0), early-(BCLC A), intermediate-(BCLC B) and advanced-(BCLC C) stage, with treatment largely stage-dependent [1-3]. As the update is fairly recent, most guidelines still use the 2011 criteria, which assesses liver function based on the Child’s score alone [3]. While BCLC does not recommended radiotherapy as a first line option throughout all stages, limitations in current modalities emphasize the importance of radiotherapy in bridging these gaps.

Curative therapies such as local ablation, surgical resection, and liver transplantation are recommended for BCLC stage 0-A patients, with 5-year overall survival (OS) of 50–70% [1-3]. As HCC is often diagnosed in the intermediate-advanced stage, only one-third are eligible for curative therapies [1]. Surgical resection is limited to patients with good liver function, missing a large proportion, as 90% of HCC arises from cirrhosis [1]. On the other hand, radiotherapy is applicable to a wider pool of patients, demonstrating efficacy and safety even in the treatment of cirrhotics [4,5].

As transplantation is limited by donor shortage, and long waiting periods leads to tumor progression and dropout [1], radiotherapy’s role in bridging and downstaging enables more patients to qualify for curative treatment. Local ablation such as radiofrequency ablation (RFA) and microwave ablation shows similar OS to resection in early-stage HCC <2 cm, but risk of local recurrence increases above 3 cm [2].

Noncurative therapies for BCLC B-C patients prolong survival and act as a bridge to transplantation. First-line options include transarterial chemoembolization (TACE) and systemic therapy with tyrosine kinase inhibitors (TKIs) [1-3]. TACE allows selective delivery of chemotherapy, but patients with impaired liver or renal function or poor portal vein (PV) blood flow are less suitable [6], making radiotherapy a useful alternative for such patients. Systemic therapy is also riddled with side effects such as hand-foot skin reactions and arterial hypertension in a dose-dependent manner [7]. Sorafenib is recommended for patients with PV tumor thrombosis (PVTT), but response rates are dismal (2–5%), and median time to progression (TTP) is 2.8 months [8]. Recently, immune checkpoint inhibitors and gene-targeted oncolytic viral therapy have an emerging role in advanced HCC [1]. For patients with unresectable HCC, the recent IMbrave150 trial showed significantly better OS and 2.5 month increase in progression free survival (PFS) with atezolizumab plus bevacizumab as compared to sorafenib [9]. The role of radiotherapy as an immunomodulator makes it especially relevant, with the potential of enhancing such new modalities in HCC treatment.

While clinicians have more options for HCC management, unmet needs are reflected through the limitations explained above, and the mixed treatment outcome for intermediate-stage HCC. While classically deemed to be radioresistant, present-day radiobiologic studies show that HCC has similar radiosensitivity to other common epithelial tumors treated with radiotherapy [10]. As HCC is radiosensitive, radiation therapies play a significant role in HCC management. Radiation therapies offer local control in unresectable lesions, palliation in metastatic disease, and a bridge to resection and transplantation in selected patients [6]. Newer radiotherapy modalities offer improved dose planning and targeted delivery, allowing for better tumor response and safer dose escalations while minimizing the risks of radiation-induced liver damage (RILD). This report aims to provide an overview of the role of radiation therapies in HCC management.

## 2. Principles of Radiotherapy

Radiosensitivity shows the likelihood of cells to be damaged by radiation by measuring the fraction of clonogens that survive a given X-ray dose [10,11]. Radiotherapy works by damaging cellular components and DNA, effectively targeting actively dividing cancer cells [11]. Hepatic nonparenchymal cells represent 30–35% of cells in the liver and include Kupffer cells, endothelial cells, fat-storing cells, and pit cells, most of which reside within hepatic sinusoids [8]. As these cells are radiosensitive, radiation exposure releases large amounts of reactive mediators, eicosanoids, proteolytic enzymes, and cytokines such as tumor necrosis factor-alpha. These hepatotoxic products promote apoptosis and fibrosis, altering the hepatic architecture with resultant hepatic dysfunction [8]. The limiting factor of hepatotoxicity necessitates a delicate balance between eradicating cancer cells and minimizing RILD [11].

Two types of RILD exist. Historically, classic RILD occurred as a complication in up to 5–10% of the patients 2 weeks–4 months after mean liver dose of 30–35 Gy is given using conventionally fractionated regimens and is thought to be due to veno-occlusive disease as a result of fibrosis [12]. As a more subacute form, this manifests as anicteric hepatomegaly, ascites and elevated alkaline phosphatase (ALP) up to 2×, while transaminases and bilirubin remain unchanged [13]. Symptoms of fatigue, abdominal pain, hepatomegaly may be noted on clinical history and examination.

However, with advancements in radiation dose planning and newer modalities of radiation delivery, non-classic RILD has become the more common manifestation and is defined as an elevation of serum transaminase (>5× upper limit of normal) and worsening of CP score ≥2 [12]. The ALP is usually normal. The non-classic variation typically develops in patients with a background of cirrhosis or viral hepatitis, and is thought to be a consequence of reactivating hepatitis and a loss of regenerating hepatocytes [12].

**Figure 1:**
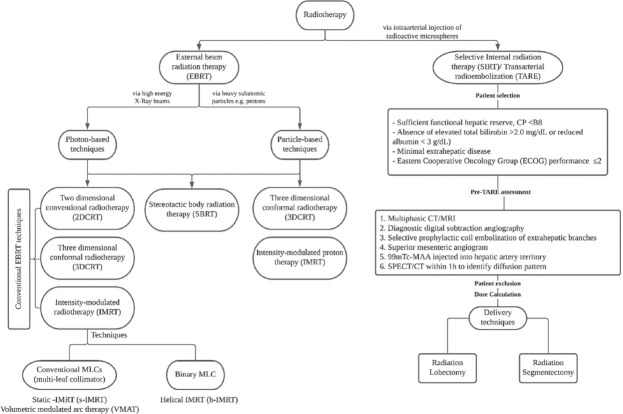
Radiotherapy modalities for HCC management. 99mTc-MAA: 99m technetium-labeled macroaggregated albumin; CT: Computerized tomography; CP: Child Pugh; GIT: Gastrointestinal tract; HCC: Hepatocellular carcinoma; MRI: Magnetic resonance imaging; SPECT/CT: Single-photon emission CT; EBRT: External beam radiation therapy; SIRT: Selective internal radiation therapy; 3DCRT: Three-dimensional conformal radiotherapy; IMRT: Intensity-modulated radiotherapy

**Figure 2:**
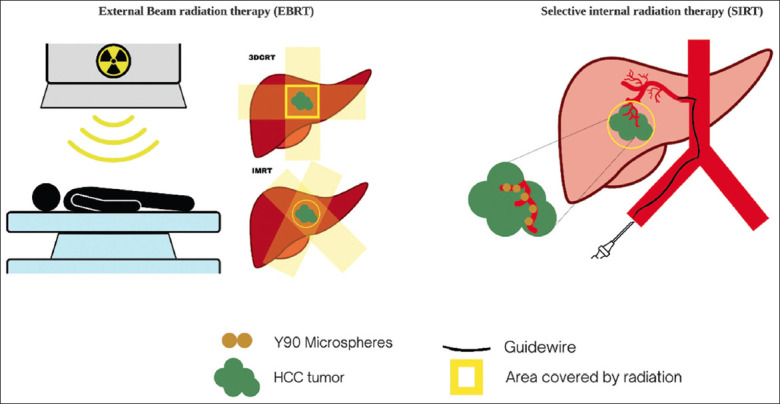
A pictorial representation of the differences between EBRT and SIRT. EBRT, external beam radiation therapy; SIRT: Selective internal radiation therapy; 3DCRT: Three-dimensional conformal radiotherapy; IMRT: Intensity-modulated radiotherapy

Radiotherapy modalities are broadly classified into external beam radiation therapy (EBRT) and selective internal radiation therapy (SIRT) (Figures 1 and 2). With emerging modalities such as image-guided radiotherapy, radiotherapy has a complementary role in the multidisciplinary care of HCC patients.

## 3. EBRT

### 3.1. Photon-based techniques

Conventional EBRT includes two-dimensional conventional radiotherapy (2DCRT), three-dimensional conformal radiotherapy (3DCRT), and intensity-modulated radiotherapy (IMRT). 2DCRT requires minimal imaging, allowing for treatment to be started earlier. However, as CT planning is not performed, gross tumor volume (GTV), clinical target volume (CTV), and internal target volume (ITV), and critical organs at risk (OARs) are not formally defined. As whole-liver tolerance radiation dose is lower than the HCC tumoricidal dose, 2DCRT has fallen out of favor [14]. 3DCRT minimizes RILD and improves objective response rates (ORR) [15,16]. CT scan also allows precise calculation of GTV, CTV, ITV, and OAR. 3DCRT results in higher response (ORR 77.1%) and mOS (13 months). However, it has side effects at high doses [17].

IMRT utilizes 3D images in an inverse treatment planning regimen and is a more advanced form of EBRT. Studies comparing IMRT to 3DCRT show that IMRT demonstrates higher local control rates (LCR), 1-year OS, and 3-year OS, with similar toxicity (RILD rate <5%) [18,19]. Higher doses resulted in superior outcomes with no significant difference in gastrointestinal tract (GIT) bleeding or RILD. Doses ≥72 Gy improve OS and surgical conversion rate [20]. In terms of critical OARs, IMRT resulted in lower mean doses to the stomach, left kidney, and small bowel than 3DCRT, with h-IMRT showing the best results [21]. However, for larger tumors (>6 cm), 3DCRT may reduce the RILD risk [22,23]. Large scale randomized controlled trials comparing these modalities are required to confirm these findings.

For intermediate HCC, guidelines recommend TACE and TKIs. EBRT shows improved mOS in patients with tumor thrombus in the portal vein (PV) branch, PV trunk, inferior vena cava (IVC), and PV+IVC [24,25]. Compared to the prognosis of 2.4–2.7 months without treatment, 6.5 months with TKIs, EBRT improve survival [26]. In a meta-analysis including patients with IVC thrombus, Chai *et al*. reported that EBRT has a LCR of 83.8% and an overall grade ≥3 complication rate of 1.2% [27]. In a separate meta-analysis comparing EBRT to surgery, mOS and 1-year OS were lower for EBRT, but 2-year OS was similar to surgery (26.9% and 27.5%, respectively) [28]. Thus, EBRT is a comparable non-invasive alternative.

In HCC patients, lymph node involvement is considered metastatic. Survival is poor and systemic therapy is the standard of care [26]. Surgical lymphadenectomy has no role due to uncontrolled primary tumor, background liver dysfunction, and concurrent distant metastasis [22]. In a meta-analysis of 8 studies comprising 521 patients, Chai *et al*. evaluated the combined utility of EBRT techniques in patients with lymph node metastasis and found that HCC patients with lymph node metastases had a 1-year OS 41.0%, with EBRT. Groups with higher radiation doses displayed better RR (82.2% vs. 51.1% in the low dose group), with low rates of grade ≥3 toxicities [23]. AASLD 2018 guidelines and EASL-European Organization for Research and Treatment of Cancer guidelines classify EBRT therapy as a low-grade recommendation based on a lack of good quality evidence. Multi-center collaborative randomized studies including a large sample of patients with clearly defined inclusion and exclusion criteria are essential to improve the scientific body of evidence.

### 3.2. Stereotactic body radiation therapy (SBRT)

SBRT is a non-invasive radiotherapy combining stereotactic technology with 3DCRT, accurately targeting the tumor’s center while drastically reducing surrounding doses. It involves delivering potentially ablative fractional doses over shorter treatment durations. Fractional doses delivered are much higher, ranging between 5 and 10 Gy compared to conventional radiotherapy (typical daily dose between 1.8 and 3Gy), allowing abbreviated treatment duration (between 1 and 2 weeks vs. 5 and 7 weeks) [6]. SBRT thus results in better dose distributions and high LCR (87–100%) [29]. However, the high doses call for increased precision, careful patient immobilization, advanced tumor tracking with daily imaging, and respiratory motion management [6].

To identify the optimal dose and fractionation regimens for SBRT, a multicenter retrospective study classified 602 patients based on the SBRT dose received. Higher doses were associated with better OS, PFS and LCR, and the following doses were recommended: Biologically effective dose (BED_10_) ≥ 100 Gy as a first-line ablative dose, or equivalent dose in 2 Gy fractions (EQD2) ≥ 74 Gy as a second-line radical dose, and EQD2 < 74 Gy as palliative irradiation [30]. In keeping the risk of RILD ≤5%, D50 (dose that would result in a 50% LC) at 6 and 9 months was 53 Gy EQD2 and 84 Gy EQD2, respectively [31]. This was slightly higher in a separate study in Korea (D50=62.9 Gy EQD2) [32]. In general, common dose regimens such as 40–48 Gy in 3 fractions and 35–40 Gy in 5 fractions can achieve a 2-year LCR of 90% [29].

While a dose-response relationship is widely-established, clinical value in terms of translation into survival advantage have been mixed, with some suggesting that a critical threshold has to be attained before OS can be improved [33]. A multicenter trial demonstrated this threshold to be BED ≥ 53 Gy10 [34]. A separate study showed that doses >54 Gy in 3 fractions (BED=152 Gy10, EQD2=126 Gy) achieved LCR of 100% with a 2-year OS of 71%, while patients receiving <45 Gy in 3 fractions (BED=113 Gy10, EQD2=94 Gy) experienced a lower 2-year LCR and OS rate (64% and 30%, respectively) [32].

A consecutive phase I to II study of 102 CP A patients treated with 6-fraction SBRT to a median total dose of 36 Gy (range: 24–54 Gy) demonstrated 1-year LCR of 87% for tumors with a median diameter of 7.2 cm [33]. A separate study including CP A and CP B7 patients displayed similar results when treated to a median total dose of 48 Gy in 3 fractions (range: 36–48) and 40 Gy in 5 fractions, respectively [35]. LCR of 91% was seen in CP A patients, but is slightly lower (82%) in CP-B7 patients. Higher rates of hepatotoxicity were seen in CP B7 patients, with 38% experiencing grade ≥3 toxicity (vs. 11% in CP A) [35]. A CP-score increase of ≥2 is associated with a 63% increased risk at 3 months [36]. Due to a relative lack of survival advantage in CP ≥B8 patients mOS 2.8 months (vs. 9.9 months in CP-B7 patients) and the high hepatotoxicity, SBRT is often avoided in patients with CP ≥B8 [36]. Other factors that may portend a poorer prognosis include the presence of PVTT, multinodular disease and high serum α-fetoprotein (AFP) >4491 ng/mL [29].

SBRT is helpful in all stages of HCC and is recommended (level 2 evidence) for patients with BCLC stage A as an alternative to thermal ablation in curative management. In early-stage unresectable HCC patients not amenable for local ablation, SBRT demonstrates improved response and survival in small HCC, with a complete response (CR) and partial response (PR) rate of 15.5% and 45.7%, respectively, and 1-year and 3-year OS rate of 86.0% and 53.8%, respectively. For HCC between 2.1–3 cm and ≤2 cm, SBRT showed a high LCR of 93.3% and 100%, respectively. However in patients with HCC>3 cm, LCR is lower (76.3%) [37].

Overall, SBRT is a valuable adjunct in patients with disease progression after liver-directed therapies. In patients with HCC <2 cm, OS is comparable to RFA, but in patients with HCC >2 cm, RFA had better OS [38]. However, for patients with localized HCC without vascular invasion and ineligible for RFA or TACE, SBRT resulted in high LCR and long-term OS, with 1-, 3- and 5-year OS 77.3%, 39.0%, and 24.1%, respectively [39]. Cox proportional hazard regression analysis also showed that post-SBRT liver transplant resulted in significantly improved OS [39].

For small HCC patients, a dose of 30 Gy/5 fractions has been determined to be safe and effective for cirrhotic patients [4]. A dose of 50 Gy/5 fractions in nonmetastatic HCC patients demonstrated good LCR (95%) and 1-year OS of 87% with only 1/9 patients with Child-Pugh ≥B8 experiencing grade ≥3 hepatic toxicity [5]. SBRT is also safe as a bridge-to-transplant and acts as a complimentary alternative to TACE and RFA, demonstrating comparable OS and dropout rates [40]. For advanced HCC, SBRT at a dose of 45 Gy/10 fractions demonstrated LCR of 91%, with 1- and 3-year OS rates of 62% and 28%, respectively [41]. In a multicenter study of patients with unresectable primary HCC, SBRT showed decreased median tumor volume (*P*<0.004), median TTP of 6.3 months, and 1- and 2-year OS of 87% and 55%, respectively [42]. Even for patients with advanced liver failure ineligible for transplant, SBRT demonstrated safety, with a mOS of 8.8 months [43].

### 3.3. Particle therapy

Particle therapy such as carbon ion therapy or proton beam therapy (PBT) involves the use of particles such as heavier charged carbon ions or protons. Unlike photon-based EBRT which involves the firing of X-ray beam multiple times from different angles, radiation delivery in particle-based EBRT occurs through particle accelerators which form a single beam of high energy protons to be delivered into the patient [44]. Its ability to provide more localized particle exposure compared to photon-based EBRTs allows for higher doses to be delivered while reducing the damage to surrounding tissues and unwanted side effects [45].

While an exponential decrease is seen in deeper tissues for conventional photon-based techniques, PBT’s finite range allows for superior dose distribution as they deliver low doses on entering the target tissue, and only show a steep maximum (Bragg-Peak) upon reaching a specific depth (dependent on their energy). Beyond this depth, there is close to no delivery of radiation, hence, majority of their dose is delivered near the end of their target range and over a narrow range, while relatively low doses occur outside the Bragg peak region [44]. 3 main delivery methods exist to allow for uniform coverage at all depths and cover the entire target volume: (1) Passive scattering, uniform scanning and active scanning [46]. Moreover, as a heavier particle, carbon ions also have the added advantage of inducing irreparable damage to DNA and are less dependent on the oxygen availability of tumor tissues, allowing for increased distribution of energy during their travel through the tissue (higher linear-energy transfer) and treatment of hypoxic tumors resistant to photons [45].

PBT protocols have been developed by the Proton Medical Research Center (PMRC) of the University of Tsukuba, Japan, with dose recommendations based on tumor location concerning porta hepatis and GIT critical OARs [47]. For peripheral tumors >2 cm away from the both the GIT and porta hepatis, 66 GyE/10 fractions is recommended, while tumors ≤2 cm of the GIT can be treated with 77.0 GyE/35 fractions and tumors ≤2 cm of the GIT can be treated with 72.6 GyE/22 fractions. These were recommended based on a LCR range of 88–95% and 3-year OS of 45–65% [48].

Evaluating the safety and effectiveness of PBT, in a phase II trial of 76 cirrhotic patients with HCC (mean size 5.5 cm), there were minimal acute toxicities and no significant difference in RILD 6-months post-treatment [49]. Patients with HCC ≤2 cm of the GIT treated with PBT at a dose of 72.6 GyE/22 fractions or 77 GyE/35 fractions had mOS of 33.9 months, a 3-year OS of 50%, and a grade 3 GIT hemorrhage risk of 2.1% [47]. In another phase II multicenter trial in unresectable HCC patients, PBT (67.5 GyE/5 fractions) showed a 2-year LCR and OS of 94.8% and 63.2% [50]. For advanced HCC with PVTT and a median tumor size of 60 mm, patients treated with PBT (median total dose 72.6 GyE in 22 fractions) had OS of 48% and 21% at 2 and 5 years, respectively, with an mOS of 22 months [51]. The national cancer center of Korea also demonstrated 2-year LCR and OS of 88.1% and 51.1%, respectively [52].

PBT is well tolerated even in large HCC, with low rates of grade ≥3 toxicities [53-55]. For HCC>10 cm, PMRC reported 1-year and 2-year OS of 64% and 36%, respectively, and 2-year LCR of 87% [56]. Even in patients with a Child’s score of C, PBT is safe. PBT not only improves LCR (95%) and 2-year OS (42%) but may also help improve liver function with better disease control [53]. Table 1 shows a comparison between PBT and photon-based techniques.

**Table 1:** A comparison between photon-based techniques and proton-based techniques

	Photon-based techniques	Particle-based techniques
Technique	Involves firing beams multiple times from different angles	Uses particle accelerators to form a single beam of high-energy protons [44]

Mechanism	Radiation delivered from an external source; dose decreases for deeper tissues	Distribution follows a Bragg-peak: Low doses delivered on entering target tissues with a steep maximum at a specific energy-dependent depth [44]

Delivery methods	2DCRT, 3DCRT, IMRT	Via heavy particles; Involves Passive scattering, uniform scanning, active scanning [46]

Comparison	Poorer OS	Better OS [54]

	Less localized radiation exposure® lower doses delivered, more collateral damage	More localized particle exposure® higher doses delivered, less collateral damage [45]

	Poorer dose distribution	Better dose distribution due to narrow Bragg-peak range [44]

	Poorer energy distribution	Better energy distribution (via higher linear-energy transfer)

	The exponential decrease in radiation as depth increase	Uniform coverage at all depths

	DNA damage may be reparable	Induce irreparable damage to DNA

	More dependent on oxygen availability ® hypoxic tumors show poorer response	Less dependent on the oxygen availability of tumor tissue® hypoxic tumors show better response [45]

2DCRT: Two-dimensional conventional radiotherapy; 3DCRT: Three-dimensional conformal radiotherapy; IMRT: Intensity-modulated radiotherapy; OS: Overall survival; DNA: Deoxyribonucleic acid

Table 2 provides an overview of the dose, toxicity profile, advantages, and limitations of all EBRTs used in the management of HCC. Table 3 summarizes the present studies showing the clinical efficacy of various EBRT techniques in patients with early-stage HCC, intermediate-stage HCC, advanced HCC, recurrent HCC, and cirrhotic patients.

**Table 2:** Comparison of EBRT modalities for HCC treatment

	2DCRT	3DCRT	IMRT	SBRT	PBT
Planning	Bony landmarks defined by X-ray [6], minimal CT required [15]	CT required [15]	4D-CT/MRI/PET [15]	CT/MRI/PET	CT/MRI/PET
Radiation beam and beam modifiers	Photons or electrons±wedge filters; coplanar beams [15]	Photons, wedges, a field in the field, compensators; several coplanar and noncoplanar beams[15]	Use of multiple modulated beamlets, Photons+IMRT, Multiple noncoplanar beams or arcs [15]; s-IMRT: Step-and-shoot and sliding window techniques; VMAT: Rotational IMRT using conventional MLCs; h-IMRT: Rotational IMRT using helical tomotherapy	Photon-based technique including radiation beams used in 3DCRT and IMRT; performed using conventional linear accelerators	Proton-based; Uses patient- and field-specific collimators, compensators, particle accelerators [46]
Total dose	<30–35 Gy [14]	45–60 Gy [14,55]	40–100 Gy [57] (customized based on GTV, ITV, PTV, CTV)	Typically 24–60 Gy [58] (determined by tumor size and OAR)	72.6Gy/22 fractions or 66Gy/10 fractions [48]
Side effects and toxicity	Highest toxicity [59]; Higher collateral dose deposition; Lowest survival and higher risk of adverse effects compared to other modalities [59]	Low toxicity [16]	No significant difference compared to 3DCRT [18,19]; Improved precision and conformality, reduced collateral dose deposition [6]; Low RILD; but higher risk of RILD for Larger tumors [17]	Low toxicity [5,37,43]	Low toxicity to liver and OARs [47,49], reduced toxicity compared to other modalities
Procedure-related	Non-invasive	Non-invasive	Non-invasive	Non-invasive More complex planning than 3DCRT, More expensive than 2D/3DCRT [6]	Non-invasive
Costs to patient	Cheapest [6]; Minimal imaging, infrastructure, and training required [6]	Inexpensive [6]; More extended treatment regimen than 2DCRT (multiple weeks) [6]	More costly with more advanced imaging requirements; More extended treatment regimen (multiple weeks), more expensive than 2D/3DCRT [6]	More costly with more advanced imaging requirements	Larger space required, more costly, limited availability, more extended treatment regimen (multiple weeks) [6]
Technical	Inadequate identification of volume (GTV, CTV, ITV) and OAR [15]	Planning requires multiple CT images [15] but better delineation of surrounding tissue than 2DCRT and collateral dose deposition; Permits targeted therapy [16]; Can compute CTV, GTV, OAR, and plan properly; Can combine stereotactic technology	Better tumor coverage More complex planning	Higher fractional doses delivered; Irradiation delivered in fewer fractions; Requires patient immobility and multi-image guidance	The dosimetric advantage compared to photon-based EBRT: Localized deposition of dose following the Bragg peak; Higher line energy transfer [44]; Increased tumor targeting, suitable in cirrhotic patients [60] Requires precise positioning of dose gradients as slight differences can lead to under/over dosage due to finite range of protons;
Efficacy and utility	Utility in resource-poor setting and emergency setting	Can treat several lesions in a single course [16]; Higher likelihood of producing a response in deeper lesions inaccessible to percutaneous procedures [16]	Improved mOS, ORR, PFS, 1-year survival rate, and LCR than 3DCRT [18,19]		Reduced efficacy with tissue heterogeneity

2DCRT: Two-dimensional conventional radiotherapy; 3DCRT: Three-dimensional conformal radiotherapy; BED: Biologically effective dose_10_; CI: Conformity index; CP: Child-Pugh classification; CT: Computerized tomography; CTV: Clinical target volume; EBRT: External beam radiation therapy; GIT: Gastrointestinal tract; GTV: Gross tumor volume; HI: Homogeneity index;

h-IMRT: Helical IMRT; IMRT: Intensity-modulated radiotherapy; ITV: Internal target volume; IVCTT: Inferior vena cava tumor thrombosis; LCR: Local control rate; MRI: Magnetic resonance imaging; MLC: Multi-leaf collimator; MVI: Macroscopic vascular invasion; OAR: Critical organs at risk; ORR: Objective response rate; OS: Overall survival; PFS: Progression-free survival;

PBT: Proton beam therapy; PVTT: Portal vein tumor thrombus; RILD: Radiation-induced liver damage; SBRT: Stereotactic body radiation therapy; s-IMRT: Static IMRT; VMAT: Volumetric modulated arc therapy

**Table 3:** Studies showing the safety and efficacy of EBRT modalities based on patient characteristics

	2DCRT	3DCRT	IMRT	SBRT	PBT
Early-stage HCC		CR 80% PR 12% [16]		mOS 15.7 months [61] 1-year OS 64.3–90.9% 2-year OS 67.5% 3-year OS 30–73.4% [61,62] 1-, 2-, 3-year LCR: 94%, 92%, 93% [62]	mOS 32.2 months 1-year OS 76.5–88.4% [61,63] 3-year OS 36.7% [61] 5-year OS 63.4% [63]

		Relapse rate 22% (similar to RFA) [64]		Comparable to thermal ablation [37]	Longer OS than SBRT [61]

Intermediate-stage HCC			3-year OS 33.4% [19]	2-year LC 87% 2-year OS 63% [32]	OS 64% PFS 62% [56]

			Longer OS than 3DCRT [19]	Bridge to transplant [40] Comparable to TACE [65]	

Advanced-stage HCC	PVTT/IVCTT: mOS 11 months, 3-year OS 20% [59] Lymph node metastasis: mOS 9.4 months [66]	PVTT/IVCTT: mOS 30 months [59] PVTT: 1-year OS 40.7–43.8% ORR of 45.8–51.3 [67,68] IVCTT: ORR 60% [69] MVI: mOS 7.9–8.8 months [70]	mOS 21 months 1-year OS 62% [20] PVC/IVCTT: mOS 30 months [59] Lymph node metastasis: RR 73.1%, 1-year OS 41.0% [23]	1-year OS 62–87% 3-year OS 28–55% [41,42] PVTT: ORR 71% [67] LCR 91% [41] FFLP 63% [42]	PVTT: 2-year LCR 88.1% 2-year OS 51.1% [52] 2-year LPFS 46% 5-year LPFS 20% [51]

			PVTT and/IVCTT: superior to 3DCRT [18] Better CI and HI than 3DCRTI [21]	Comparable to TACE [65] Higher ORR than EBRT and SIRT [67]	

Recurrent HCC/Repeat irradiation		Repeat RT: mOS 30 months [71]	Repeat RT: mOS 30 months[71] Post-hepatectomy [72,73]: 3-year OS 67.7–89.1% 1-year RFS 86.2% 2-year RFS 70.5% 3-year RFS 60.1–64.2% 1- year OS 96.6%, 2-year OS 80.7%	Post-TACE: 6 months ORR 84.8% 1- year OS 75.8% 2-year OS 45.5% mOS 19 months [74] Repeat SBRT: 3-year OS 61.0% [75]	Repeat PBT LC 87.8% OS 55.6% [60] mOS 61 months 2- year OS 87.5% 5-year OS 49.4% [76]

					Non-inferior to RFA [77] Repeat PBT safe, no acute toxicity/RILD [60,76]

Cirrhotic		CP A and B 1-year OS 65% 2-year OS 43% 3-year OS 33% mOS 20 months [78] Grade ≥3 toxicity 18.5% [16]	CP A and B mOS 12.6 months 1-year OS 56.2% 2-year OS 31.7%59 CR 5.2% PR 47.4%	CP A 1- year OS 92% 2-year OS 60% mOS 41 months 1- year LCR 82% 2-year LCR 62% CP B and C ORR 36.6–80% mOS 8.8–46 months Grade ≥3 toxicity 10% median TTP 9.7-months [43]	CP A mOS 34 months CP B mOS 13 months CP C mOS 12–17 months50 LCR 95% 1-year OS 53% 2-year OS 42%56

		CP A and B: RILD 15% [78]	CP A and B: Grade ≥3 liver toxicity 13.2% [57]	CP A, B, C: No grade ≥3 toxicity [43]	CP C: No grade ≥3 toxicities [53]

^99^mTc-MAA: 99m technetium-labeled macroaggregated albumin; CT: Computerized tomography; CR: Complete response; FFLP: Freedom from local progression; FLR: Future liver remnant ratio; GIT: Gastrointestinal tract; IVCTT: Inferior vena cava tumor thrombus; LPFS: Local progression-free survival; LCR: Local control rate; MRI: Magnetic resonance imaging; mOS: Median OS; ORR: Overall response rate; PFS: Progression-free survival; PVE: Portal vein embolization; PVTT: Portal vein tumor thrombus; PBT: Proton beam therapy; SPECT/CT: Single-photon emission CT; TD: Tumor dose; TTP: Time to progression; TTST: Time to secondary therapy

### 3.4. RT in the palliative setting

For patients with advanced stage unresectable HCC, best supportive care (BSC) is often the treatment of choice and includes analgesics for pain management. However, symptoms such as abdominal discomfort, pain, nausea or fatigue are still often reported. Low-dose RT has proven to be useful in such settings, with a dose of 8 Gy in a single fraction demonstrating a symptomatic improvement in 48% at 1 month [79]. Similarly, in a separate study evaluating the use of single dose palliative RT (8 Gy in a single fraction) in symptomatic unresectable HCC patients with an index symptom of either pain or abdominal discomfort, 51.9% demonstrated clinical improvement of their index symptom at 1 month, with the treatment being well tolerated with minimal toxicities [80]. Apart from the single fraction dose, RT can also be given in 2 fractions over 2 days (10 Gy in total), with symptomatic improvement in 53–66% at 2 weeks and minimal toxicities seen [81].

## 4. SIRT

SIRT, also known as transarterial radioembolization, involves injecting radioactive microspheres of yttrium-90 (Y90), Lipiodol labeled with iodine-131 or rhenium-188 intra-arterially [82]. The most popular technique uses Y90, a ß-emitting isotope. At present, AASLD 2018 recommends SIRT as an alternative therapy to the various modalities used in BCLC stage A, B, and C patients (level 2 and 3 evidence), while EASL 2018 states that more data from randomized controlled trials is required [2,3]. The Asian Pacific Association for the study of the Liver (APASL) recommends SIRT in patients ineligible for TACE [83]. Increased adoption of SIRT is seen with emerging data showing SIRT as comparable to current modalities. Notably, the SARAH (SorAfenib Versus Radioembolization in Advanced HCC) trial, a randomized controlled phase III trial involving 467 patients with locally advanced (BCLC C) or intermediate-stage HCC (BCLC B) who failed two rounds of TACE, showed no significant difference in OS (mOS 8.0 vs. 9.9 months in sorafenib) [84]. In Asia, another large phase III trial of 360 patients with locally advanced unresectable HCC randomized to sorafenib or SIRT demonstrated no significant difference in mOS (8.8 months in SIRT vs. 10.0 months in sorafenib). However, patients with SIRT experienced fewer grade ≥3 adverse effects (*P*<0.001), demonstrating superior toxicity profiles [85]. Further large-scale randomized controlled trials are required to support its use.

The process of SIRT is summarized in Figure 1. Sufficient hepatic reserve is required due to the risk of liver failure. Pre-SIRT assessment involves a multiphasic Computerized tomography (CT)/Magnetic resonance imaging (MRI) to identify the disease extent and location. Digital subtraction angiography outlines the foregut vascular anatomy, while prophylactic embolization of extrahepatic branches reduces spillage of microspheres into GIT [86]. Superior mesenteric angiogram determines the variant vessels to the liver, with delayed images helping to assess PV patency. This is followed by injecting 99m technetium-labeled macroaggregated albumin (^99m^Tc-MAA) into the hepatic artery territory, acting as a surrogate for Y90 microspheres. Finally, SPECT/CT is performed within 1h to identify diffusion patterns which will help predict the subsequent distribution of microspheres [87]. This also enables physicians to establish appropriate entry points for the catheter, assess hepatopulmonary shunting, and detect GIT deposition [88]. Patients with the following are not suitable: hepatopulmonary shunt fraction >20% (risk of radiation pneumonitis), and vascular abnormalities that cannot be corrected by embolization or catheter repositioning (risk of GIT toxicity) [88].

Dose calculation is performed based on quantitative analysis of the ^99m^Tc-MAA SPECT/CT. TD 205 Gy predicted response (sensitivity 100%, accuracy 91%) [89]. Patients treated with TD>205 Gy demonstrated longer TTP 13.0 months and mOS 23.2 months (vs. TD<205 Gy, which demonstrated TTP 5.5 months and mOS 11.5 months) [90]. However, as MAA is a mere surrogate, it cannot predict actual Y90 activity. SIRT may be conducted in the form of radiation segmentectomy or radiation lobectomy. Radiation segmentectomy involves transarterial infusion of microspheres into a segmental vessel. This results in radioembolization of ≤2 hepatic segments, with the intention of segmental ablation while sparing other segments [91,92]. Patients who may be suitable include those who are ineligible for surgical resection, ablation, or undergoing evaluation for liver transplantation [92]. Patients who underwent radiation segmentectomy had ORR 59% (WHO criteria) and 81% (EASL criteria), median TTP 13.6 months, and mOS 26.9 months, with minimal amounts of grade 3/4 toxicities (9%) and no RILD [91]. Compared to TACE, it improved LCR 92% and CR (92.1%) with no significant difference in OS [93]. On the other hand, radiation lobectomy involves transarterial lobar infusion of microspheres. Similar to PVE, it results in a “lobar atrophy–hypertrophy complex,” with ablation of the entire lobe and concomitant hypertrophy of the nonradiated lobe due to redirected blood flow [94]. Ipsilateral lobar atrophy reduces micro-and macro-vascular spread, while contralateral lobe hypertrophy reduces liver dysfunction risk. Volumetric changes such as liver fibrosis or portal hypertension have no clinical sequelae [95]. This is ideal for patients with unilobar tumor and preserved liver function and can be used as a primary treatment modality or as a bridge to resection or transplantation [92]. 52% of patients who underwent radiation lobectomy had a reduction in ipsilateral lobar volume, 5-year OS 46% (comparable to curative resection), and no hepatic insufficiency or major adverse effects observed [96,97].

Results from studies evaluating the SIRT are tabulated in Table 4. Compared to other curative treatments with 5-year OS rates between 60 and 80% in BCLC stage 0 and stage A HCC patients [98], SIRT provides comparable outcomes, acting as an alternative for patients who are ineligible for curative treatment. SIRT also has a role in bridging (treatment for waiting list patients within transplant criteria) or downstaging (reduce tumor burden for patients within transplant criteria) for transplantation. A retrospective study showed SIRT the highest CR rate of 75% (vs. TACE 41%, RFA 60%, SBRT 28.5%) [99]. Another study reported that none of the 15 patients on SIRT progressing from UNOS T2 to T3 stage, and 8/10 patients downstaged from T3 to T2 [100]. Complete tumor necrosis was seen in 47% of HCCs ≤ 5 cm [101]. Table 5 compares SIRT to present treatment modalities.

**Table 4:** Summary of studies investigating the safety and efficacy of SIRT

Independent studies							

Study	Total	CP	ECOG	PVTT	Extra-hepatic involvement	Tumor characteristic	Safety and Efficacy
Kulik *et al*. 2008 [102]	*n*=108	A/B/C=54/27/1	0–2	37	13	Unresectable Intermediate-advanced	PR: WHO 42.2%, EASL 70% No treatment-related complications or deaths
Mazzaferro *et al*. 2013 [103]	*n*=52	A-B7	0–1	35	None	Intermediate-advanced	mOS=15 months ORR=40.4% TTP=11 months
Salem *et al*. 2010 [104]	*n*=291	A=131 B=152 C=8	0–2	125	46	All stages	ORR: WHO=42%, EASL=57% TTP=7.9 months mOS: CP A/B=17.2/7.7 months
Sangro *et al*. 2011 [105]	*n*=325	A=268 B=57	0–3	76	30	All stages	mOS=12.8 months (BCLC A, B, C=24.4, 16.9, 10 months)
Lewandowski *et al*. 2018 [106]	*n*=70	A	NA	0	0	Early-stage PVTT: absent	RR 6-months=EASL (86%), WHO (49%) TTP=2.4 years

**Meta-analysis**							

Lobo *et al*.[107] 2016	*n*=553 (5 comparative studies, with quality assessed by the STROBE criteria)			CR and PR: No significant difference (vs cTACE) Vs. cTACE: Less post-treatment pain, more subjective fatigue; no difference in nausea, vomiting, fever, or other complications			
Massani *et al*. 2016 [108]	*n*=1431 (8 studies)			OS: No significant difference (vs. TACE) Adverse events: Less than TACE			
Yang *et al*. 2018 [96]	*n*=1652 (11 studies, including 2 RCTs)			OS: Increased 2-year OS OR: Better (vs. TACE, mRECIST criteria) Adverse events: Less than cTACE			
Gardini *et al*. 2018 [109]	*n*=97 (3 RCTs)			OS, PFS: No significant difference at 1-year Bridging: Higher proportion underwent transplant			

c-TACE: Conventional TACE; CP: Child-Pugh; DEB-TACE: Drug-eluting bead TACE; ECOG: Eastern Co-operative Oncology Group performance status; ORR: Objective response rates; OS: Overall survival; PVTT: Portal vein tumor thrombosis; RCT: Randomized controlled trials; RR: Response rate: SIRT: Selective internal radiation therapy; TTP: Time to progression; STROBE: Strengthening the Reporting of Observational studies in Epidemiology criteria

**Table 5:** Comparison between SIRT and TKIs or TACE, respectively

Population	Both used as a noncurative treatment for HCC patients with BCLC stage B-C	Wider patient pool; Suitable for patients with more advanced liver disease, multifocal disease, vascular invasion, and PVTT [97]
Intervention	SIRT	SIRT
Comparator	TKI	TACE
Outcome	SIRT compared with other modalities	
Safety and Side effects	Side effects less common [84,85]	Better toxicity profile [97], less PES [110]; Less post-treatment pain, more subjective fatigue, no difference in nausea, vomiting, fever, or other complications [107]
Adverse events/complications	Less common [111], less grade 3/4 adverse events requiring dose modifications or interruptions	Less adverse events [96,108,112]
OS, PFS	No significant difference [84,85,111,113,114]	No significant difference in OS [108,109,115]; OS and PFS at 1-year: No significant difference [109]; Better 2- and 3-year OS (vs. cTACE), more inferior 2-year OS (vs. DEB-TACE) [112]
TTP	No significant difference [111,114]	Longer [110] median TTP (>26 months vs. 7 months) [115]; No significant difference [116]
Response	Higher ORR [84]	EASL: No significant difference [115]; Response rate (CR, PR): No significant difference [107]; Better ORR [96]
Bridging	SIRT allows for bridging to curative treatment	Bridging for transplantation: Greater tumor shrinkage [117], higher proportion proceed to transplant [109], higher response [99]
Other considerations	More significant cost savings (5.4–24.9%) [118]	Shorter hospitalization, can perform outpatient [110] Fewer treatment sessions [109,110], higher pre-treatment cost [119], less cost-effective in BCLC Stage A-B but more cost-effective in BCLC-C [119]; Quality of life: FACT-Hep scores similar [120] but better performance in sub-features of quality of life [121]

CR: Complete response; EASL: European Association for the Study of the Liver; FACT-Hep: Functional Assessment of Cancer Therapy-Hepatobiliary; ORR: Objective response rates; OS: Overall Survival; PFS: Progression-free survival; PES: Post-embolization syndrome; PR: Partial response; PVTT: Portal vein tumor thrombus; SIRT: Selective internal radiation therapy; TACE: tranSarterial chemoembolization; TKI: Tyrosine kinase inhibitors; TTP: time To progression; WHO: World Health Organization

## 5. Assessment of Response

An accurate evaluation of treatment response is essential for clinical surveillance and prognosis, and assessing the tumor may be objectively determined based on various criteria (Table 6). Radiation success can generally be divided into technical and clinical success. Most frameworks assess technical success, with changes in tumor size being the primary biomarker. Clinical success is often neglected. As radiotherapy causes tumor de-vascularization, cavitation, and necrosis changes, which may not affect tumor size (size reduction occurring gradually over 4–6 months), treatment response may be underestimated [122]. Moreover, reduction in enhancement precedes the decrease in size [123], and a paradoxical increase may also occur due to intra-tumoral hemorrhage, edema, and necrosis [95].

**Table 6:** Comparison of criteria measuring response to treatment*

	Complete response	Partial response	Progressive disease
Tumor size (%)			
WHO [124]	Disappearance of all lesions	≥50% ↓	≥25% ↑
RECIST [136]	The disappearance of all lesions	≥30% ↓ in the sum of diameters	≥20% ↑
mRECIST [137]	The disappearance of intratumoral arterial enhancement in all lesions	≥30% ↓ in the sum of diameters of viable (enhance in arterial phase) target lesions	≥20% ↑in the sum of diameters of viable (enhancing) target lesions
Choi [138]	The disappearance of all lesions	≥10% ↓ OR≥15% ↓ in tumor density (CT)	≥10% ↑ and Tumor density does not meet PR criteria
Modified Choi [137]	The disappearance of all lesions	≥10% ↓ AND≥15% ↓ in tumor density (CT)	
Non-target Lesions			
WHO	The disappearance of all lesions	-	≥1
RECIST	The disappearance of all lesions	Present	Unequivocal progression
mRECIST	The disappearance of intratumoral arterial enhancement	Intratumoral arterial enhancement in≥1 lesion	Unequivocal progression
Choi	The disappearance of all lesions	No obvious progression of non-measurable disease	New intratumoral nodules/↑ size of existing nodules
Modified Choi	The disappearance of all lesions	No obvious progression of non-measurable disease	New intratumoral nodules/↑ size of existing nodules
New lesions			
WHO	-	-	≥1 new lesion
RECIST	-	-	≥1 new lesion
mRECIST	-	-	≥1 new lesion
Choi	-	-	≥1 new lesion
Modified Choi	-	-	≥1 new lesion
Clinical	Choi and Modified Choi: SD- No symptomatic deterioration caused by tumor progression *Clinical symptoms are not accounted for in other criteria*		
Overall response			
WHO	Poorest response designation used		
RECIST	Result of the combined assessment of target lesions, non-target lesions, and new lesions		
mRECIST	Result of the combined assessment of target lesions, non-target lesions, and new lesions		
Choi	Responders: ≥10% decrease in tumor size OR≥15% decrease in tumor density on CT		
	Non-responders: Do not meet the above criteria		
Modified Choi	Responders: ≥10% decrease in tumor size OR≥15% decrease in tumor density on CT		

* Stable Disease (SD) refers to any lesions that do not qualify under the criteria of CR/PR/PD. CT: Computed tomography; mRECIST: Modified Response evaluation criteria in solid tumors; RECIST: Response evaluation criteria in solid tumors; WHO: World Health Organization

According to the WHO, the overall response is categorized into four groups: CR, PR, stable disease (SD), and progressive disease (PD), based on imaging findings [124]. As the response is based on the measurement of viable tumors, this provides a better indication of OS than total tumor measurement, as it involves identifying areas with treatment-induced necrosis [125]. Patients with an objective response (CR or PR) as determined by mRECIST had longer OS than non-responding patients (SD or PD) (18 months vs. 8 months, *P*=0.013) [126]. Furthermore, in patients with SD as identified by RECIST, OS also differed depending on their tumor response based on mRECIST, with patients who achieved CR, PR, and SD having a median OS of 17 months, 10 months, and 4 months respectively (*P*=0.016) [126]. EASL measures response differently: CR (absence of enhancing tissue), PR (>50% decrease in enhancing tissue), SD (<50% decrease in enhancing tissue), PD (increase in the enhancement of treated tumor that translates into additional locoregional therapy).

CT is the primary modality for HCC imaging in both the diagnostic and follow-up phases. In addition, Quadriphase MDCT can be done to characterize residual enhancement [127]. Dual-energy CT helps detect HCC and evaluate response to locoregional therapy [128]. Bremsstrahlung SPECT/CT and positron emission tomography (PET)/CT determine the safe distribution of Y90 microspheres, and the presence of aberrant microsphere deposition that may help predict side effects [129]. However, as lesions may undergo coagulative necrosis and internal hemorrhage post-radiotherapy, MRI’s ability to obtain subtraction images where the native T1 signal is cancelled makes it easier to distinguish hemorrhage from enhancement, ensuring a high accuracy [130]. 18F-Fluorodeoxyglucose-PET (18F-FDG PET) uptake reflects tissue metabolism and is associated with treatment response. A decrease in standardized uptake values (SUV) ratio post-EBRT correlates with the degree of tumor necrosis on histological examination, and EBRT patients with higher SUV ratios displayed higher response rates [131]. Pre-operative FDG predicts risk of recurrence post-surgery [132]. FDG post-TACE displayed higher diagnostic accuracy over triphasic CT [133] and contrast-enhanced CT [134], but its utility post-radiotherapy has not been validated.

Assessment time depends on imaging modality and institution guidelines, but tumor response to radiotherapy often shows more gradual changes such as reduced enhancement and size over several months. Standard recommendations for frequency and time of assessment are not present in current guidelines, but post-SIRT imaging usually occurs at 1 month and every 2–3 months after that, with a higher frequency in the 1^st^ year due to a 6.5× higher risk recurrence compared to the 2^nd^ year [135]. Boas *et al*. suggest a schedule of 8 time points in the first 2 years (2, 4, 6, 8, 11, 14, 18, and 24 months) as this reduces diagnostic delay and is cost-effective [135]. Our practice is to follow-up patients every 3 months with a multiphasic CT scan or an MRI liver.

## 6. Side Effects

Common side effects of radiotherapy include nausea, vomiting, fatigue, diarrhea, and loss of appetite. These are usually mild and self-limiting. SIRT side effects can result from embolic effects of microspheres and are termed post-radioembolization syndrome (PRS), occurring in 20–70% [139]. Symptoms usually last a few hours, and hospitalization is often not required [140]. Patients often experience mild symptoms that are less severe than other embolic therapies [140] (e.g., fatigue [54–61%], abdominal pain [23–56%], nausea and vomiting [20–32%], and low-grade fever [3–12%]) [140]. As PRS is expected, patients should be pre-empted, and appropriate pharmaco-prophylaxis administered. Lymphopenia may be seen but is not associated with increased infection risk [141].

## 7. Complications

Traditionally, radiotherapy’s utility has been limited as doses >30 Gy run high risks of RILD. RILD may occur acutely, within the first few weeks of radiotherapy or up to years later, but typically presents within the first 4–8 weeks; hence, vigilant follow-up is necessary during this period. The lack of effective treatment to prevent or cure RILD makes it particularly problematic, and close monitoring of liver function aids early diagnosis. Pre-clinical measurement of liver volume and CP scores helps predict RILD. Most patients that develop RILD have a CP score>6 and should be watched with caution as recovery from RILD is poor in this group [142]. Thankfully, risks of RILD have decreased with good patient selection, improved image guidance, and targeted delivery. Patients with liver dysfunction have low tolerance, requiring dose reduction. Child’s A and B patients treated with >50 Gy and <50 Gy radiation witnessed 8.4% and 5.3% RILD, respectively [55]. Doses >100 Gy are associated with low RILD risk when the irradiated liver volume is <20% [143]. Similar to the need to preserve an adequate future liver remnant post-hepatectomy, a critical minimum volume of 700 cc of liver should be spared by SBRT (by receiving <15 Gy) given the importance of ensuring sufficient liver function [144].

Radioembolization-induced liver disease (REILD) occurs specifically in SIRT and refers to symptomatic ascites or jaundice within 8 weeks post-SIRT in the absence of tumor progression or biliary obstruction. REILD is associated with an elevated bilirubin (>3 mg/dL) and variable GGT and ALP increases. Risk factors include (1) exposure to chemotherapy within 2-months post-SIRT, (2) small liver (total volume <1.5 L), (3) high baseline bilirubin and aspartate aminotransferase, (4) repeated whole-liver SIRT [145]. However, REILD incidence is reducing with refinement in dosimetry and patient selection [146].

## 8. Comparisons among Radiotherapy Modalities

Comparing the radiotherapy modalities in treating advanced HCC with PVTT, a meta-analysis showed SIRT and SBRT to have no significant difference in 1- and 2-year OS, but patients with SBRT demonstrated the highest response rate (71% vs. 51% in EBRT and 33% in SIRT) [67]. In cirrhotic patients, EBRT affects functional hepatic reserve. Hence, SIRT is preferred [147]. For PVTT patients, pooled response rates and 1-year OS were higher in 3DCRT and SBRT than in SIRT (51%, 71%, and 33%, respectively) [67]. However, in unresectable HCC, no significant difference in OS or disease-specific survival was seen [148].

## 9. Combination Therapy

Patients with unresectable early-stage HCC are commonly treated with TACE, but recurrence is common and side effects from repeated TACE such as liver and renal failure are debilitating. Combination therapies have been studied to mitigate this and help improve patient outcomes. Combining EBRT with SIRT showed 1-, 2-, and 3-year OS rates of 59.8%, 47.9%, and 47.9%, respectively. 36% developed grade >2 liver toxicities. However, restricting the dosage reduces the likelihood of hepatotoxicity (*P*=0.03) [149]. Patients treated with 3DCRT and TACE had OS rates significantly higher than patients treated with each modality alone (*P*<0.05) [150]. A meta-analysis on 3DCRT with TACE also demonstrated superiority compared to TACE monotherapy for patients with advanced HCC, resulting in higher 1-, 2- and 3-year OS (Odds ratio [OR]=1.87, 2.38 and 2.97, respectively), higher tumor response (OR=3.81) and decline in AFP (OR=3.24). For patients with PVTT, ORR was highest in the combined group (50% vs. 35.3% and 29.2% in patients treated with 3DCRT or TACE monotherapy, respectively), but differences were not significant. mOS and OS at 1, 2, and 3 years were significantly higher in the combined group (13 months, 53.5%, 18,8%, and 9.4%, respectively) [150].

Comparing SBRT to TACE, a propensity-score matched analysis demonstrated comparable LCR with no significant difference in OS [65]. Furthermore, even when SBRT was combined with TACE in small HCC, the SBRT-TACE group had similar outcomes to SBRT monotherapy (2-year OS 80% vs. 79% for SBRT alone, PFS 43% vs. 49% SBRT alone) [43]. In another retrospective study for patients with early-stage HCC ineligible for resection or ablation, no significant difference in treatment results or toxicity was seen for SBRT monotherapy versus SBRT-TACE combination [151]. However, another propensity score analysis in HCC patients with PVTT demonstrated significant improvement in survival when TACE was combined with SBRT (10.9 months vs. 4.1 months for patients treated with TACE alone) [152].

In unresectable HCC, IMRT following TACE achieved ORR 64.8%, mOS 20.2 months, PFS 10.5 months, and actuarial 1-, 2-, and 3-year OS rates of 84.6%, 49.7%, and 36.7%, respectively. In terms of safety, 18.5% developed grade 3 hematological toxicity while 5.6% developed grade 3 hepatic toxicity, and none experienced grade 4 or 5 toxicity [153]. For the newer PBT, in a randomized trial of HCC patients who met the Milan or San Francisco liver transplant criteria, preliminary results favored PBT, with higher pathologic CR (25% vs. 10%), 2-year LCR (88% vs. 45%), and PFS (48% vs. 31%). However, as results were not statistically significant, we await further results on completing this trial [154].

## 10. Future Development

Several trials investigating the role of radiotherapy in HCC management are underway. As PVTT involvement is commonly seen in HCC, a study done in Guangxi province, China (NCT04025437) sought to determine the safety and efficacy of neoadjuvant radiotherapy for HCC involving type I PVTT given the high 5 year recurrence rate of up to 75% post-hepatectomy. Examining the utility of combination treatment, a phase II clinical trial (NCT03535259) studied the safety and efficacy of combining IMRT with sorafenib in treatment of patients with advanced HCC. IMRT is given to the hepatic primary tumor, vein tumor thrombosis, and metastasis lymph node, in conjunction with a 400mg twice daily dose of sorafenib simultaneously. RT alone treatment gives a response of 50–60%, with high incidences of out RT field failure in the form of liver and distance metastasis while sorafenib alone treatment response rate is low (2–5%) [8,11]. We await results from this study in determining the utility of combining both modalities to achieve a synergistic effect. In the palliative setting, RT has also been investigated as a complimentary modality to BSC in the alleviation of pain (NCT02511522).

While curative hepatectomy is the standard treatment of choice for HCC patients with adequate liver function, high rates of intrahepatic recurrences post-resection (70–100% after 5 years) make adjuvant radiotherapy increasingly relevant [73]. Risk factors for post-operative recurrence include: Tumor size (especially >5 cm), number, and histopathological grade; microvascular invasion (MVI) and macrovascular invasion; presence of stellate nodules, underlying liver disease, and surgical factors (extent of resection and resection margins) [155]. MVI is the most commonly reported and is an independent prognostic factor associated with early postoperative recurrence and poor OS. While a resection margin of 2 cm has been deemed to be safe in reducing post-operative recurrence, cirrhotic patients often have limited liver reserves. Adjuvant radiotherapy is a promising adjunct, resulting in significantly longer recurrence-free survival and OS in patients with MVI, as compared to TACE [73].

Radiotherapy is also beneficial in patients with close surgical margins (<1 cm). Patients with positive margin resection who underwent adjuvant SBRT had lower rates of total recurrence (22.2% vs. 65.1% for patients with narrow-margin resection without SBRT and 44.0% in patients with wide-margin resection) [156]. For centrally located HCC, adjuvant 3DCRT after narrow-margin hepatectomy did not show a significant difference in OS but demonstrated safety, with no cases of RILD observed [157]. IMRT also displayed favorable outcomes for patients with narrow-margin resection, with 3-year OS comparable to patients with wide-margin hepatectomy (89.1% vs. 86.0%, respectively). Patients who underwent adjuvant IMRT also had significantly better 3-year OS (*P*=0.009), fewer early recurrences (*P*=0.002), and fewer extrahepatic metastases (*P*=0.038) compared to those with narrow margin resection who did not undergo adjuvant IMRT [72].

Radiotherapy also offers a possibility of downstaging when used as a neoadjuvant treatment. Compared to surgery alone, patients treated with neoadjuvant 3DCRT had significantly improved survival outcomes (1-year OS 75.2% vs. 43.1% for hepatectomy-alone patients) and lower recurrence rates, attributed to the decrease in tumor volume and downstaging of the PVTT type following neoadjuvant radiotherapy [158]. As interleukin (IL-6) levels were significantly higher in pre-radiotherapy serum and tumor tissues of non-responders, overexpression of IL-6 may be signal a poorer prognosis [158]. A retrospective analysis of 244 patients also showed neoadjuvant radiotherapy to be superior to post-operative radiotherapy, with a significant improvement in OS seen [159].

Recent studies investigating the use of mesenchymal stem cells (MSC) in mitigating radiotoxicity show promise. MSC infusion has facilitated recovery post-irradiation and was associated with decreased liver transaminases and inhibition of apoptosis in animal models [160]. Anti-inflammatory and immune-modulatory properties of MSC and MSC-derived bioactive components also inhibit fibrosis and enhance angiogenesis, stimulating reparative processes and providing a protective effect against RILD [161]. In rats pre-treated with intravenous MSC-conditioned medium (MSC-CM) immediately before receiving liver irradiation, anti-apoptotic effects were observed in sinusoidal endothelial cells. MSC-CM also reduced the secretion and expression of inflammatory cytokines while increasing anti-inflammatory cytokines, suggesting its role in preventing RILD [161].

Combination treatment with immunotherapy is gaining interest as radiotherapy’s utility extends beyond its cytotoxic effects. In terms of tumor control, its ability to modulate the immune microenvironment suggests potential combination therapies with immune checkpoint inhibitors. Radiotherapy works by four key steps, inducing: (1) Antigen release and immunogenic cell death, (2) antigen-presenting cell maturation and antigen presentation, (3) T-cell recruitment and infiltration, and (4) tumor-cell sensitization to immune-mediated cell death. Blocking co-stimulatory and inhibitory signals that allow for tumor immune resistance presents a synergistic effect with immune checkpoint inhibitors [162]. Kim *et al*. also demonstrated superior anti-tumor effects when radiotherapy was combined with anti-PD-L1 in murine models, demonstrating significant improvement in survival compared to both groups alone (*P*<0.01), attributing this to the upregulation of PD-L1 expression in tumor cells through the Interferon-γ/signal transducer and activator of transcription 3 signalling [163].

Although the efficacy of immune checkpoint inhibitors efficacy in treating HCC is dismal (<20% response rate), combining it with a tumor microenvironment-modulator allowed it to perform better than sorafenib, as seen in the IMbrave150 trial, where atezolizumab and bevacizumab were used [9]. In addition, OS and PFS at 12 months were higher in the combination group (OS 67.2% vs. 54.6%, PFS 6.8 vs. 4.3 months) while toxicity was similar. However, clinical studies combining radiotherapy and immune checkpoint inhibitors in HCC treatment are lacking. For example, Chiang *et al*. reported ORR 100% in 5 patients treated with sizeable unresectable HCC [164]. Furthermore, Tai *et al*. showed in a phase II trial of 36 patients that combining SIRT with nivolumab gave an ORR of 31%, with only 11% experiencing grade 3/4 toxicities [165]. Finally, results from trials elucidating the efficacy of combining radiotherapy with immunotherapy are pending. A phase II trial combining pembrolizumab and radiotherapy (NCT03316872) is estimated to complete in 2022.

HCC management is evolving. Twenty-two clinical practice guidelines are reported in the 3 years from 2018 to 2020. Table 7 shows the recommendations for radiotherapy from current guidelines, with most suggesting it as an alternative due to a lack of quality evidence. Unlike western guidelines which recommend radiotherapy as alternative options to current modalities for patients with different stages of HCC, the latest 2021 Japanese guidelines did not mention the use of radiotherapy, and instead recommends hepatic artery infusion chemotherapy and immunotherapy as alternative options, with increased focus on the use of immunotherapy [166]. However, the 2018 Korean guidelines align more closely to Western guidelines, recommending the use of EBRT in combination with or as an alternative to TACE, and as a palliative treatment modality. SIRT is also a possible alternative to TACE [167].

**Table 7:** Summary of recommendations regarding radiotherapy from present guidelines

Guidelines	Year	Recommendations regarding radiotherapy
2018 KLCSG–NCC Practice Guidelines for the Management of HCC [167]	2019	mUICC stage I: EBRT as an alternative option mUICC stage II: - Single ≥ 2 cm: SIRT and EBRT as alternative option - Single ≤ 2 cm: EBRT as 1^st^ line option - Multiple ≤ 2 cm: EBRT as an alternative option if tumor number ≤ 3 mUICC stage III - Single ≤ 2 cm: TACE + EBRT as 1^st^ line, EBRT as alternative option - Multiple ≤ 2 cm: TACE + EBRT as 1^st^ line - Multiple 2–3 cm: EBRT as an alternative option of tumor number ≤ 3 mUICC stage Iva - Multiple ≤ 2 cm: TACE + EBRT as 1^st^ line - Node + but no metastasis: EBRT as an alternative option - Metastasis: EBRT as an alternative option
2019 Update of INASL Consensus on Prevention, Diagnosis, and Management of HCC in India: The Puri II Recommendations [169]	2019	SIRT - Indicated in a select group of patients with advanced HCC, e.g., patients with PVTT with good liver function (CP A) - In patients suitable for both TACE and SIRT, TACE is preferred SBRT - BCLC stage B: Option for residual or recurrent lesions after TACE as part of combination therapy - BCLC stage C with thrombus involving the main portal vein: SBRT followed by sorafenib is an option
AASLD guidelines for the treatment of HCC [170], AASLD guidelines for the Diagnosis, Staging, and Management of HCC [2]	2018	- Adults with cirrhosis and HCC (T2 or T3, no vascular involvement) who are not candidates for resection or transplantation): SIRT (very low evidence), EBRT (very low evidence) - SBRT: An alternative to thermal ablation for BCLC A - SIRT: An alternative for BCLC A and B patients - For adults with cirrhosis and HCC (T2–3, no vascular involvement) who are not candidates for resection or transplantation: SIRT as an alternative (quality evidence very low)
Argentinian CPG for surveillance, diagnosis, staging, and treatment of HCC [171]	2020	SIRT - Insufficient evidence to recommend or suggest SIRT over TACE as 1^st^ option for BCLC–B patients (quality of evidence low to very low) - In some patients with large unresectable tumors, with portal vein obstruction SIRT may have a therapeutic role. (quality of evidence low) - It is uncertain to recommend or suggest SIRT after TACE failure in BCLC–B (quality of evidence high) - SIRT is not recommended for BCLC–B patients with tumor progression or BCLC–C patients (with vascular invasion) over sorafenib (quality of evidence high) - There is no recommendation to support the combination of SIRT with sorafenib for BCLC–B patients to avoid tumor progression (quality of evidence high) SBRT - SBRT is not recommended as a first–line option but is uncertain as a second option
SBH updated recommendations for diagnosis and treatment of HCC [172]	2020	SIRT (Moderate level of evidence; weak recommendation) - Promising therapeutic option with a good safety profile - Intermediate HCC: Insufficient data favoring SIRT over TACE for patients - Advanced HCC (BCLC C): Insufficient data favoring SIRT over sorafenib - The subgroup of patients who would benefit needs to be better defined
EASL Clinical Practice Guidelines: Management of HCC [173]	2018	- EBRT: No robust evidence to support this therapeutic approach in the management of HCC (Evidence low, recommendation weak) - SIRT good safety profile and local tumor control, but the subgroup of patients benefitting from SIRT needs to be defined (evidence moderate)
NCCN guidelines version 5.2020 Hepatobiliary Cancers [174]	2020	Locoregional therapy (e.g., EBRT, SIRT) as an option for - HCC is potentially resectable or transplantable, operable by performance status or comorbidity - HCC unresectable, non–transplant candidate - Liver–confined disease, inoperable by performance status, comorbidity, or with minimal or uncertain extrahepatic disease
NCCN guidelines version 5.2020 Hepatobiliary Cancers [174]	2020	EBRT - Hypofractionation with photons/protons is acceptable for intrahepatic tumors, though treatment at centers with experience is recommended - Palliative option for symptom control and prevention of complications from metastatic HCC - Dosing: Initial volumes to 45 Gy in 1.8Gy per fraction SBRT - Alternative to ablation/embolization or when these therapies fail or are contraindicated - For patients with 1–3 tumors - Consider for larger lesions of more extensive disease if there is sufficient uninvolved liver and liver radiation tolerance acceptable - Dosing: 30–50 Gy (typically in 3–5 fractions)
HCC: ESMO Clinical Practice Guidelines for diagnosis, treatment, and follow–up [175]	2018	BCLC 0-A: SBRT and SIRT as an alternative treatment (Level III evidence) BCLC B: SIRT as an option for patient’s refractory to TACE or who failed TACE (Level III evidence) BCLC: SIRT as an alternative for patients with the liver confined disease, good liver function, and who has not undergone systemic therapy (Level III evidence)
Management consensus guideline for HCC: 2020 update on surveillance, diagnosis, and systemic treatment by the TLCA and GEST [176]	2020	HCC with no extrahepatic spread/vascular invasion, CP A/B patient - 0–3 nodules: EBRT as alternative - 2–3 nodules, >3 cm: EBRT and SIRT as alternative - ≥4 nodules: SIRT as alternative HCC with no extrahepatic spread/vascular invasion, CP C patient within transplant criteria - EBRT as bridging therapy HCC with no extrahepatic spread but with vascular invasion, CP A/B patient - TACE in combination with EBRT or SIRT
Nonsurgical management of advanced HCC: A CPG [177]	2020	SIRT/SBRT - Intermediate/advanced HCC: Insufficient evidence for the use of SIRT or SBRT
Pan–Asian adapted ESMO CPG for the management of patients with intermediate and advanced/relapsed HCC: A TOS–ESMO initiative endorsed by CSCO, ISMPO, JSMO, KSMO, MOS and SSO [178]	2020	SIRT - Alternative to TACE as first–line therapy for patients with intermediate or advanced stage HCC without the extrahepatic disease (level III evidence, Grade C recommendation) - Alternative for TACE–failed BCLC B or non–metastatic BCLC C HCC patients (level III evidence, Grade C recommendation)
SASLT practice guidelines on the diagnosis and management of HCC [179]	2020	SIRT - Bridging for transplant: Alternative form of locoregional therapy - BCLC B: An alternative to TACE for patients with intermediate–stage HCC associated with portal vein thrombosis (Weak recommendation, low–quality evidence) SBRT - Alternative to RFA in patients with larger tumors (>2 cm) or tumors in a challenging location

AASLD: American Association for the Study of Liver Diseases; CP: Child–Pugh; CPG: Clinical Practice Guidelines; CSCO: Chinese Society of Clinical Oncology; EASL: European Association for the Study of the Liver; EBRT: External beam radiation therapy; ESMO: European Society for Medical Oncology; GEST: Gastroenterological Society of Taiwan; HCC: Hepatocellular carcinoma; INASL: Indian National Association for Study of the Liver; ISMPO: Indian Society of Medical and Pediatric Oncology; IVC: inferior vena cava; JSMO: Japanese Society of Medical Oncology; KLCSG–NCC: Korean Liver Cancer Study Group (KLCSG)–National Cancer Center (NCC); KSMO: Korean Society of Medical Oncology; MOS: Malaysia Oncological Society; mUICC: Modified Union for International Cancer Control; PVTT: Portal vein tumor thrombus; RFA: Radiofrequency ablation; SBRT: Stereotactic body radiation therapy; SIRT: Selective internal radiation therapy; SASLT: Saudi Association for the Study of Liver Diseases and Transplantation; SBH: Brazilian Society of Hepatology; SSO: Singapore Society of Oncology; TACE: transarterial chemoembolization; TLCA: Taiwan Liver Cancer Association; TOS: Taiwan Society for Oncology

Hence, while the progress in radiotherapy is heartening, further quality research involving larger sample sizes and reduced heterogeneity is needed before radiotherapy is advocated as a curative adjunct. As BCLC has acted as a cornerstone for several guidelines mentioned above, evidence-based updates regarding the role of radiotherapy based on substantiation by robust evidence is necessary to guide physicians for the optimal treatment of HCC patients.

The present coronavirus disease of the 2019 (COVID-19) epidemic has also influenced the management of HCC. The 2020 APASL provides recommendations for radiotherapy based on weighing the benefits of treatment and the risks from the novel coronavirus infection. For patients with low risk of progression, or those treated palliatively as a form of symptom control, the radiotherapy schedule should be delayed. However, for patients with rapidly progressing HCC, radiotherapy outweighs the risks of the COVID infection, and for function- or life-threatening situations, for example, spinal cord compression and IVC syndrome, radiotherapy treatment should proceed without delay. However, the course of radiation should be shortened [168].

## 11. Conclusion

Radiotherapy has evolved as a treatment modality, with increasing evidence demonstrating its safety and utility in the management of HCC. This is especially relevant for patients with unresectable tumors. Further research focusing on improving the precision of radiation delivery for both EBRT and SIRT, as well as quality evidence from well-designed studies will allow a personalized approach to HCC management.

## References

[1] Forner A, Reig M, Bruix J (2018). Hepatocellular Carcinoma. Lancet.

[2] Marrero JA, Kulik LM, Sirlin CB, Zhu AX, Finn RS, Abecassis MM (2018). Diagnosis, Staging, and Management of Hepatocellular Carcinoma:2018 Practice Guidance by the American Association for the Study of Liver Diseases. Hepatology.

[3] Dufour JF, Greten TF, Raymond E, Roskams T, De T (2012). Clinical Practice Guidelines EASL EORTC Clinical Practice Guidelines:Management of Hepatocellular Carcinoma European Organisation for Research and Treatment of Cancer. J Hepatol.

[4] Takeda A, Oku Y, Sanuki N, Kunieda E, Koike N, Aoki Y (2012). Dose Volume Histogram Analysis of Focal Liver Reaction in Follow-up Multiphasic CT Following Stereotactic Body Radiotherapy for Small Hepatocellular Carcinoma. Radiother Oncol.

[5] Baumann BC, Wei J, Plastaras JP, Lukens JN, Damjanov N, Hoteit M (2018). Stereotactic Body Radiation Therapy (SBRT) for Hepatocellular Carcinoma. Am J Clin Oncol Cancer Clin Trials.

[6] Chen CP (2020). Role of External Beam Radiotherapy in Hepatocellular Carcinoma. Clin Liver Dis.

[7] Raoul JL, Adhoute X, Penaranda G, Perrier H, Castellani P, Oules V (2019). Sorafenib:Experience and Better Manage-ment of Side Effects Improve Overall Survival in Hepatocellular Carcinoma Patients:A Real-Life Retrospective Analysis. Liver Cancer.

[8] Laskin DL (1991). Parenchymal and Nonparenchymal Cell Interactions in Hepatotoxicity. In:Advances in Experimental Medicine and Biology.

[9] Finn RS, Qin S, Ikeda M, Galle PR, Ducreux M, Kim TY (2020). Atezolizumab Plus Bevacizumab in Unresectable Hepatocellular Carcinoma. N Engl J Med.

[10] Wigg AJ, Palumbo K, Wigg DR (2010). Radiotherapy for Hepatocellular Carcinoma:Systematic Review of Radiobiology and Modeling Projections Indicate Reconsideration of its Use. J Gastroenterol Hepatol.

[11] Brown AP, Constine LS (2012). Radiation Therapy Principles. Am Cancer Soc.

[12] Koay EJ, Owen D, Das P (2018). Radiation-induced Liver Disease and Modern Radiotherapy. Semin Radiat Oncol.

[13] Kim J, Jung Y (2017). Radiation-induced Liver Disease:Current Understanding and Future Perspectives. Exp Mol Med.

[14] Rim CH, Yoon WS (2018). Leaflet Manual of External Beam Radiation Therapy for Hepatocellular Carcinoma:A Review of the Indications, Evidences, and Clinical Trials. Onco Targets Ther.

[15] (2008). International Atomic Energy Agency:Transition from 2-D Radiotherapy to 3-D Conformal and Intensity Modulated Radiotherapy, Iaea-Tecdoc-1588.

[16] Mornex F, Girard N, Beziat C, Kubas A, Khodri M, Trepo C (2006). Feasibility and Efficacy of High-dose Three-dimensional-conformal Radiotherapy in Cirrhotic Patients with Small-size Hepatocellular Carcinoma Non-eligible for Curative Therapies-Mature Results of the French Phase II RTF-1 Trial. Int J Radiat Oncol Biol Phys.

[17] Chen D, Wang R, Meng X, Liu T, Yan H, Feng R (2014). A Comparison of Liver Protection among 3-D Conformal Radiotherapy, Intensity-modulated Radiotherapy and RapidArc for Hepatocellular Carcinoma. Radiat Oncol.

[18] Hou JZ, Zeng ZC, Wang BL, Yang P, Zhang JY, Mo HF (2016). High Dose Radiotherapy with Image-guided Hypo-IMRT for Hepatocellular Carcinoma with Portal Vein and/or Inferior Vena Cava Tumor Thrombi is more Feasible and Efficacious than Conventional 3D-CRT. Jpn J Clin Oncol.

[19] Yoon HI, Lee IJ, Han KH, Seong J (2014). Improved Oncologic Outcomes with Image-guided Intensity-Modulated Radiation Therapy Using Helical Tomotherapy in Locally Advanced Hepatocellular Carcinoma. J Cancer Res Clin Oncol.

[20] Byun HK, Kim HJ, Im YR, Kim DY, Han KH, Seong J (2019). Dose Escalation by Intensity Modulated Radiotherapy in Liver-directed Concurrent Chemoradiotherapy for Locally Advanced BCLC Stage C Hepatocellular Carcinoma. Radiother Oncol.

[21] Lee IJ, Seong J, Koom WS, Kim YB, Jeon BC, Kim JH (2011). Selection of the Optimal Radiotherapy Technique for Locally Advanced Hepatocellular Carcinoma. Jpn J Clin Oncol.

[22] Sun HC, Zhuang PY, Qin LX, Ye QH, Wang L, Ren N (2007). Incidence and Prognostic Values of Lymph Node Metastasis in Operable Hepatocellular Carcinoma and Evaluation of Routine Complete Lymphadenectomy. J Surg Oncol.

[23] Rim CH, Kim CY, Yang DS, Yoon WS (2018). The Role of External Beam Radiotherapy for Hepatocellular Carcinoma Patients with Lymph Node Metastasis:A Meta-analysis of Observational Studies. Cancer Manag Res.

[24] Hou JZ, Zeng ZC, Zhang JY, Fan J, Zhou J, Zeng MS (2012). Influence of Tumor Thrombus Location on the Outcome of External-beam Radiation Therapy in Advanced Hepatocellular Carcinoma with Macrovascular Invasion. Int J Radiat Oncol Biol Phys.

[25] Yu J Il, Park HC, Lim DH, Park W, Yoo BC, Paik SW (2011). Prognostic Index for Portal Vein Tumor Thrombosis in Patients with Hepatocellular Carcinoma Treated with Radiation Therapy. J Korean Med Sci.

[26] Cheng AL, Kang YK, Chen Z, Tsao CJ, Qin S, Kim JS (2009). Efficacy and Safety of Sorafenib in Patients in the Asia-Pacific Region with Advanced Hepatocellular Carcinoma:A Phase III Randomised, Double-blind, Placebo-controlled Trial. Lancet Oncol.

[27] Rim CH, Kim CY, Yang DS, Yoon WS (2018). External Beam Radiation Therapy to Hepatocellular Carcinoma Involving Inferior Vena Cava and/or Right Atrium:A Meta-analysis and systemic Review. Radiother Oncol.

[28] Lee HA, Park S, Seo YS, Yoon WS, Shin IS, Rim CH (2020). Surgery Versus External Beam Radiotherapy for Hepatocellular Carcinoma Involving The inferior Vena Cava or Right Atrium:A Systematic Review and Meta-analysis. medRxiv.

[29] Schaub SK, Hartvigson PE, Lock MI, Høyer M, Brunner TB, Cardenes HR (2018). Stereotactic Body Radiation Therapy for Hepatocellular Carcinoma:CurrentTrends and Controversies. Technol Cancer Res Treat.

[30] Su TS, Liu QH, Zhu XF, Liang P, Liang SX, Lai L (2021). Optimal Stereotactic Body Radiotherapy Dosage for Hepatocellular Carcinoma:A Multicenter Study. Radiat Oncol.

[31] Lausch A, Sinclair K, Lock M, Fisher B, Jensen N, Gaede S (2013). Determination and Comparison of Radiotherapy Dose Responses for Hepatocellular Carcinoma and Metastatic Colorectal Liver Tumours. Br J Radiol.

[32] Jang WI, Kim MS, Bae SH, Cho CK, Yoo HJ, Seo YS (2013). High-dose Stereotactic Body Radiotherapy Correlates Increased Local Control and Overall Survival in Patients with Inoperable Hepatocellular Carcinoma. Radiat Oncol.

[33] Bujold A, Massey CA, Kim JJ, Brierley J, Cho C, Wong RK (2013). Sequential Phase I and II Trials of Stereotactic Body Radiotherapy for Locally Advanced Hepatocellular Carcinoma. J Clin Oncol.

[34] Seong J, Lee IJ, Shim SJ, Lim DH, Kim TH, Kim JH (2009). A Multicenter Retrospective Cohort Study of Practice Patterns and Clinical Outcome on Radiotherapy for Hepatocellular Carcinoma in Korea. Liver Int.

[35] Lasley FD, Mannina EM, Johnson CS, Perkins SM, Althouse S, Maluccio M (2015). Treatment Variables Related to Liver Toxicity in Patients with Hepatocellular Carcinoma, Child-Pugh Class A and B Enrolled in a Phase 1-2 Trial of Stereotactic Body Radiation Therapy. Pract Radiat Oncol.

[36] Culleton S, Jiang H, Haddad CR, Kim J, Brierley J, Brade A (2014). Outcomes Following Definitive Stereotactic Body Radiotherapy for Patients with Child-Pugh B or C Hepatocellular Carcinoma. Radiother Oncol.

[37] Yoon SM, Lim YS, Park MJ, Kim SY, Cho B, Shim JH (2013). Stereotactic Body Radiation Therapy as an Alternative Treatment for Small Hepatocellular Carcinoma. PLoS One.

[38] Rajyaguru DJ, Borgert AJ, Smith AL, Thomes RM, Conway PD, Halfdanarson TR (2018). Radiofrequency Ablation Versus Stereotactic Body Radiotherapy for Localized Hepatocellular Carcinoma in Nonsurgically Managed Patients:Analysis of the National Cancer Database. J Clin Oncol.

[39] Mathew AS, Atenafu EG, Owen D, Maurino C, Brade A, Brierley J (2020). Long Term Outcomes of Stereotactic Body Radiation Therapy for Hepatocellular Carcinoma without Macrovascular Invasion. Eur J Cancer.

[40] Sapisochin G, Barry A, Doherty M, Fischer S, Goldaracena N, Rosales R (2017). Stereotactic Body Radiotherapy vs. TACE or RFA as a Bridge to Transplant in Patients with Hepatocellular Carcinoma. An Intention-to-treat Analysis. J Hepatol.

[41] Chan LC, Chiu SK, Chan SL (2011). Stereotactic Radiotherapy for Hepatocellular Carcinoma:Report of a Local Single-centre Experience. Hong Kong Med J.

[42] Ibarra RA, Rojas D, Snyder L, Yao M, Fabien J, Milano M (2012). Multicenter Results of Stereotactic Body Radiotherapy (SBRT) for Non-resectable Primary Liver Tumors. Acta Oncol (Madr).

[43] Qiu H, Moravan MJ, Milano MT, Usuki KY, Katz AW (2018). SBRT for Hepatocellular Carcinoma:8-Year Experience from a Regional Transplant Center. J Gastrointest Cancer.

[44] Bodine EN, Moniay KL (2017). A Proton Therapy Model Using Discrete Difference Equations with an Example of Treating Hepatocellular Carcinoma. Math Biosci Eng.

[45] Rackwitz T, Debus J (2019). Clinical Applications of Proton and Carbon Ion Therapy. Semin Oncol.

[46] McGowan SE, Burnet NG, Lomax AJ (2013). Treatment Planning Optimisation in Proton Therapy. Br J Radiol.

[47] Nakayama H, Sugahara S, Fukuda K, Abei M, Shoda J, Sakurai H (2011). Proton Beam Therapy for Hepatocellular Carcinoma Located Adjacent to the Alimentary Tract. Int J Radiat Oncol Biol Phys.

[48] Mizumoto M, Okumura T, Hashimoto T, Fukuda K, Oshiro Y, Fukumitsu N (2011). Proton Beam Therapy for Hepatocellular Carcinoma:A Comparison of Three Treatment Protocols. Int J Radiat Oncol Biol Phys.

[49] Bush DA, Kayali Z, Grove R, Slater JD (2011). The Safety and Efficacy of High-dose Proton Beam Radiotherapy for Hepatocellular Carcinoma:A Phase 2 Prospective Trial. Cancer.

[50] Hong TS, Wo JY, Yeap BY, Ben-Josef E, McDonnell EI, Blaszkowsky LS (2016). Multi-institutional Phase II Study of High-dose Hypofractionated Proton Beam Therapy in Patients with Localized, Unresectable Hepatocellular Carcinoma and Intrahepatic Cholangiocarcinoma. J Clin Oncol.

[51] Sugahara S, Nakayama H, Fukuda K, Mizumoto M, Tokita M, Abei M (2009). Proton-beam Therapy for Hepatocellular Carcinoma Associated with Portal Vein Tumor Thrombosis. Strahlenther Onkol.

[52] Kim DY, Park JW, Kim TH, Kim BH, Moon SH, Kim SS (2017). Risk-adapted Simultaneous Integrated Boost-proton Beam Therapy (SIB-PBT) for Advanced Hepatocellular Carcinoma with Tumour Vascular Thrombosis. Radiother Oncol.

[53] Hata M, Tokuuye K, Sugahara S, Fukumitsu N, Hashimoto T, Ohnishi K (2006). Proton Beam Therapy for Hepatocellular Carcinoma Patients with Severe Cirrhosis. Strahlenther Onkol.

[54] Sanford NN, Pursley J, Noe B, Yeap BY, Goyal L, Clark JW (2019). Protons Versus Photons for Unresectable Hepatocellular Carcinoma:Liver Decompensation and Overall Survival. Int J Radiat Oncol Biol Phys.

[55] Park HC, Seong J, Han KH, Chon CY, Moon YM, Suh CO (2002). Dose-response Relationship in Local Radiotherapy for Hepatocellular Carcinoma. Int J Radiat Oncol Biol Phys.

[56] Sugahara S, Oshiro Y, Nakayama H, Fukuda K, Mizumoto M, Abei M (2010). Proton Beam Therapy for Large Hepatocellular Carcinoma. Int J Radiat Oncol Biol Phys.

[57] Huang CM, Huang MY, Tang JY, Chen SC, Wang LY, Lin ZY (2015). Feasibility and Efficacy of Helical Tomotherapy in Cirrhotic Patients with Unresectable Hepatocellular Carcinoma. World J Surg Oncol.

[58] Zeng ZC, Seong J, Yoon SM, Cheng JC, Lam KO, Lee AS (2017). Consensus on Stereotactic Body Radiation Therapy for Small-Sized Hepatocellular Carcinoma at the 7^th^ Asia-Pacific Primary Liver Cancer Expert Meeting. Liver Cancer.

[59] Kong XQ, Dong YP, Wu JX, He JY, Le YY, Du KX (2017). High-biologically Effective Dose Palliative Radiotherapy for a Tumor Thrombus Might Improve the Long-term Prognosis of Hepatocellular Carcinoma:A Retrospective Study. Radiat Oncol.

[60] Hashimoto T, Tokuuye K, Fukumitsu N, Igaki H, Hata M, Kagei K (2006). Repeated Proton Beam Therapy for Hepatocellular Carcinoma. Int J Radiat Oncol Biol Phys.

[61] Hasan S, Abel S, Verma V, Webster P, Arscott WT, Wegner RE (2019). Proton Beam Therapy Versus Stereotactic Body Radiotherapy for Hepatocellular Carcinoma:Practice Patterns, Outcomes, and the Effect of Biologically Effective Dose Escalation. J Gastrointest Oncol.

[62] Dobrzycka M, Spychalski P, Rostkowska O, Wilczyński M, Kobiela P, Grąt M (2019). Stereotactic Body Radiation Therapy for Early-stage Hepatocellular Carcinoma a Systematic Review on Outcome. Acta Oncol (Madr).

[63] Lin SY, Chen CM, Huang BS, Lai YC, Pan KT, Lin SM (2021). A Preliminary Study of Hepatocellular Carcinoma Post Proton Beam Therapy Using MRI as an Early Prediction of Treatment Effectiveness. PLoS One.

[64] Lin SM, Lin CJ, Lin CC, Hsu CW, Chen YC (2004). Radiofrequency Ablation Improves Prognosis Compared with Ethanol Injection for Hepatocellular Carcinoma ≤4 cm. Gastroenterology.

[65] Bettinger D, Gkika E, Schultheiss M, Glaser N, Lange S, Maruschke L (2018). Comparison of Local Tumor Control in Patients with HCC Treated with SBRT or TACE:A Propensity Score Analysis. BMC Cancer.

[66] Zeng ZC, Tang ZY, Fan J, Qin LX, Ye SL, Zhou J (2005). Consideration of Role of Radiotherapy for Lymph Node Metastases in Patients with HCC:Retrospective Analysis for Prognostic Factors from 125 Patients. Int J Radiat Oncol Biol Phys.

[67] Rim CH, Kim CY, Yang DS, Yoon WS (2018). Comparison of Radiation Therapy Modalities for Hepatocellular Carcinoma with Portal Vein Thrombosis:A Meta-analysis and Systematic Review. Radiother Oncol.

[68] Dae YK, Park W, Do HL, Lee JH, Yoo BC, Paik SW (2005). Three-dimensional Conformal Radiotherapy for Portal Vein Thrombosis of Hepatocellular Carcinoma. Cancer.

[69] Kouloulias V, Mosa E, Georgakopoulos J, Platoni K, Brountzos I, Zygogianni A (2013). Three-dimensional Conformal Radiotherapy for Hepatocellular Carcinoma in Patients Unfit for Resection, Ablation, or Chemotherapy:A Retrospective Study. Sci World J.

[70] Asagi A, Ishii H, Uesugi K, Nadano S, Kajiwara T, Tanimizu M (2016). Three-dimensional Conformal Radiotherapy (3D-CRT) for Macroscopic Vascular Invasion (MVI) of Advanced Hepatocellular Carcinoma (HCC). J Clin Oncol.

[71] Huang Y, Chen PY, Cheng TY, Chiou JF (2021). Hepatic Reirradiation for Patients with Recurrent Hepatocellular Carcinoma. Appl Sci.

[72] Wang WH, Wang Z, Wu JX, Zhang T, Rong WQ, Wang LM (2015). Survival Benefit with IMRT Following Narrow-margin Hepatectomy in Patients with Hepatocellular Carcinoma Close to Major Vessels. Liver Int.

[73] Wang L, Wang W, Rong W, Li Z, Wu F, Liu Y (2020). Postoperative Adjuvant Treatment Strategy for Hepatocellular Carcinoma with Microvascular Invasion:A Non-randomized Interventional Clinical Study. BMC Cancer.

[74] Yao E, Chen J, Zhao X, Zheng Y, Wu X, Han F (2018). Efficacy of Stereotactic Body Radiotherapy for Recurrent or Residual Hepatocellular Carcinoma after Transcatheter Arterial Chemoembolization. Biomed Res Int.

[75] Kimura T, Takeda A, Tsurugai Y, Kawano R, Doi Y, Oku Y (2020). A Multi-Institutional Retrospective Study of Repeated Stereotactic Body Radiation Therapy for Intrahepatic Recurrent Hepatocellular Carcinoma. Int J Radiat Oncol Biol Phys.

[76] Oshiro Y, Mizumoto M, Okumura T, Fukuda K, Fukumitsu N, Abei M (2017). Analysis of Repeated Proton Beam Therapy for Patients with Hepatocellular Carcinoma. Radiother Oncol.

[77] Kim TH, Koh YH, Kim BH, Kim MJ, Lee JH, Park B (2021). Proton Beam Radiotherapy vs. Radiofrequency Ablation for Recurrent Hepatocellular Carcinoma:A Randomized Phase III Trial. J Hepatol.

[78] Liang SX, Zhu XD, Lu HJ, Pan CY, Li FX, Huang QF (2005). Hypofractionated Three-dimensional Conformal Radiation Therapy for Primary Liver Carcinoma. Cancer.

[79] Soliman H, Ringash J, Jiang H, Singh K, Kim J, Dinniwell R (2013). Phase II Trial of Palliative Radiotherapy for Hepatocellular Carcinoma and Liver Metastases. J Clin Oncol.

[80] Yeung CS, Chiang CL, Wong NS, Ha SK, Tsang KS, Ho CH (2020). Palliative Liver Radiotherapy (RT) for Symptomatic Hepatocellular Carcinoma (HCC). Sci Rep.

[81] Bydder S, Spry NA, Christie DR, Roos D, Burmeister BH, Krawitz H (2003). A Prospective Trial of Short-fractionation Radiotherapy for the Palliation of Liver Metastases. Australas Radiol.

[82] Kallini JR, Gabr A, Salem R, Lewandowski RJ (2016). Transarterial Radioembolization with Yttrium-90 for the Treatment of Hepatocellular Carcinoma. Adv Ther.

[83] Omata M, Cheng AL, Kokudo N, Kudo M, Lee JM, Jia J (2017). Asia-Pacific Clinical Practice Guidelines on the Management of Hepatocellular Carcinoma:A 2017 Update. Hepatol Int.

[84] Vilgrain V, Pereira H, Assenat E, Guiu B, Ilonca AD, Pageaux GP (2017). Efficacy and Safety of Selective Internal Radiotherapy with Yttrium-90 Resin Microspheres Compared with Sorafenib in Locally Advanced and Inoperable Hepatocellular Carcinoma (SARAH):An Open-Label Randomised Controlled Phase 3 Trial. Lancet Oncol.

[85] Chow PK, Gandhi M, Tan SB, Khin MW, Khasbazar A, Ong J (2018). SIRveNIB:Selective Internal Radiation Therapy Versus Sorafenib in Asia-Pacific Patients with Hepatocellular Carcinoma. J Clin Oncol.

[86] Covey AM, Brody LA, Maluccio MA, Getraidman GJ, Brown KT (2002). Variant Hepatic Arterial Anatomy Revisited:Digital Subtraction Angiography Performed in 600 Patients. Radiology.

[87] Ilhan H, Goritschan A, Paprottka P, Jakobs TF, Fendler WP, Bartenstein P (2015). Systematic Evaluation of Tumoral 99mTc-MAA Uptake Using SPECT and SPECT/CT in 502 Patients before 90Y Radioembolization. J Nucl Med.

[88] Bester L, Meteling B, Boshell D, Chua TC, Morris DL (2014). Transarterial Chemoembolisation and Radioembolisation for the Treatment of Primary Liver Cancer and Secondary Liver Cancer:A Review of the Literature. J Med Imaging Radiat Oncol.

[89] Garin E, Lenoir L, Rolland Y, Edeline J, Mesbah H, Laffont S (2012). Dosimetry Based on 99mTc-Macroaggregated Albumin SPECT/CT Accurately Predicts Tumor Response and Survival in Hepatocellular Carcinoma Patients Treated with 90Y-Loaded Glass Microspheres:Preliminary Results. J Nucl Med.

[90] Garin E, Lenoir L, Edeline J, Laffont S, Mesbah H, Porée P (2013). Boosted Selective Internal Radiation Therapy with 90Y-loaded Glass Microspheres (B-SIRT) for Hepatocellular Carcinoma Patients:A New Personalized Promising Concept. Eur J Nucl Med Mol Imaging.

[91] Riaz A, Gates VL, Atassi B, Lewandowski RJ, Mulcahy MF, Ryu RK (2011). Radiation Segmentectomy:A Novel Approach to Increase Safety and Efficacy of Radioembolization. Int J Radiat Oncol Biol Phys.

[92] Malhotra A, Liu DM, Talenfeld AD (2019). Radiation Segmentectomy and Radiation Lobectomy:A Practical Review of Techniques. Tech Vasc Interv Radiol.

[93] Padia SA, Johnson GE, Horton KJ, Ingraham CR, Kogut MJ, Kwan S (2017). Segmental Yttrium-90 Radioembolization Versus Segmental Chemoembolization for Localized Hepatocellular Carcinoma:Results of a Single-Center, Retrospective, Propensity Score-Matched Study. J Vasc Interv Radiol.

[94] Gaba RC, Lewandowski RJ, Kulik LM, Riaz A, Ibrahim SM, Mulcahy MF (2009). Radiation Lobectomy:Preliminary Findings of Hepatic Volumetric Response to Lobar Yttrium-90 Radioembolization. Ann Surg Oncol.

[95] Singh P, Anil G (2014). Yttrium-90 Radioembolization of Liver Tumors:What do the Images tell Us?. Cancer Imaging.

[96] Yang Y, Si T (2018). Yttrium-90 Transarterial Radioembolization Versus Conventional Transarterial Chemoembolization for Patients with Hepatocellular Carcinoma:A Systematic Review and Meta-analysis. Cancer Biol Med.

[97] Rahman SIU, Nunez-Herrero L, Berkes JL (2020). Position 2:Transarterial Radioembolization Should Be the Primary Locoregional Therapy for Unresectable Hepatocellular Carcinoma. Clin Liver Dis.

[98] Bruix J, Reig M, Sherman M (2016). Evidence-Based Diagnosis, Staging, and Treatment of Patients with Hepatocellular Carcinoma. Gastroenterology.

[99] Mohamed M, Katz AW, Tejani MA, Sharma AK, Kashyap R, Noel MS (2015). Comparison of Outcomes between SBRT, Yttrium-90 Radioembolization, Transarterial Chemoembolization, and Radiofrequency Ablation as Bridge to Transplant for Hepatocellular Carcinoma. Adv Radiat Oncol.

[100] Riaz A, Kulik L, Lewandowski RJ, Ryu RK, Spear GG, Mulcahy MF (2009). Radiologic-pathologic Correlation of Hepatocellular Carcinoma Treated with Internal Radiation Using Yttrium-90 Microspheres. Hepatology.

[101] Molvar C, Lewandowski R (2015). Yttrium-90 Radioembolization of Hepatocellular Carcinoma-Performance, Technical Advances, and Future Concepts. Semin Intervent Radiol.

[102] Kulik LM, Carr BI, Mulcahy MF, Lewandowski RJ, Atassi B, Ryu RK (2008). Safety and Efficacy of 90Y Radiotherapy for Hepatocellular Carcinoma with and without Portal Vein Thrombosis. Hepatology.

[103] Mazzaferro V, Sposito C, Bhoori S, Romito R, Chiesa C, Morosi C (2013). Yttrium-90 Radioembolization for Intermediate-advanced Hepatocellular Carcinoma:A Phase 2 Study. Hepatology.

[104] Salem R, Lewandowski RJ, Mulcahy MF, Riaz A, Ryu RK, Ibrahim S (2010). Radioembolization for Hepatocellular Carcinoma Using Yttrium-90 Microspheres:A Comprehensive Report of Long-term Outcomes. Gastroenterology.

[105] Sangro B, Carpanese L, Cianni R, Golfieri R, Gasparini D, Ezziddin S (2011). Survival after Yttrium-90 Resin Microsphere Radioembolization of Hepatocellular Carcinoma Across Barcelona Clinic Liver Cancer Stages:A European evaluation. Hepatology.

[106] Lewandowski RJ, Gabr A, Abouchaleh N, Ali R, Al Asadi A, Mora RA (2018). Radiation Segmentectomy:Potential Curative Therapy for Early Hepatocellular Carcinoma. Radiology.

[107] Lobo L, Yakoub D, Picado O, Ripat C, Pendola F, Sharma R (2016). Unresectable Hepatocellular Carcinoma:Radioembolization Versus Chemoembolization:A Systematic Review and Meta-analysis. Cardiovasc Intervent Radiol.

[108] Massani M, Pirozzolo G, Maccatrozzo P, Pozza FD, Barbisan D, Bonariol R (2016). Comparison of Transarterial Chemo-embolization (TACE) and Transarterial Radio-embolization (TARE) for the Treatment of Hepatocellular Carcinoma:A Systematic Review and Meta-analysis. HPB.

[109] Gardini AC, Tamburini E, Iñarrairaegui M, Frassineti GL, Sangro B (2018). Radioembolization Versus Chemoembolization for Unresectable Hepatocellular Carcinoma:A Meta-analysis of Randomized Trials. Onco Targets Ther.

[110] Salem R, Lewandowski RJ, Kulik L, Wang E, Riaz A, Ryu RK (2011). Radioembolization Results in Longer Time-to-progression and Reduced Toxicity Compared with Chemoembolization in Patients with Hepatocellular Carcinoma. Gastroenterology.

[111] Lemieux S, Buies A, Turgeon AF, Hallet J, Daigle G, Côté F (2021). Effect of Yttrium-90 Transarterial Radioembolization in Patients with Nonsurgical Hepatocellular Carcinoma:A Systematic Review and Meta-analysis. PLoS One.

[112] Yang B, Liang J, Qu ZY, Yang FY, Liao ZY, Gou HF (2020). Transarterial Strategies for the Treatment of Unresectable Hepatocellular Carcinoma:A Systematic Review. PLoS One.

[113] Pollock RF, Brennan VK, Shergill S, Colaone F (2021). A Systematic Literature Review and Network Meta-analysis of First-line Treatments for Unresectable Hepatocellular Carcinoma Based on Data from Randomized Controlled Trials. Expert Rev Anticancer Ther.

[114] Cho YY, Lee M, Kim HC, Chung JW, Kim YH, Gwak GY (2016). Radioembolization is a Safe and Effective Treatment for Hepatocellular Carcinoma with Portal Vein Thrombosis:A Propensity Score Analysis. PLoS One.

[115] Salem R, Gordon AC, Mouli S, Hickey R, Kallini J, Gabr A (2016). Y90 Radioembolization Significantly Prolongs Time to Progression Compared with Chemoembolization in Patients with Hepatocellular Carcinoma. Gastroenterology.

[116] Pitton MB, Kloeckner R, Ruckes C, Wirth GM, Eichhorn W, Wörns MA (2015). Randomized Comparison of Selective Internal Radiotherapy (SIRT) Versus Drug-Eluting Bead Transarterial Chemoembolization (DEB-TACE) for the Treatment of Hepatocellular Carcinoma. Cardiovasc Intervent Radiol.

[117] Sacco R, Mismas V, Marceglia S, Romano A, Giacomelli L, Bertini M (2015). Transarterial Radioembolization for Hepatocellular Carcinoma:An Update and Perspectives. World J Gastroenterol.

[118] Pollock RF, Colaone F, Guardiola L, Shergill S, Brennan VK (2020). A Cost Analysis of SIR-Spheres Yttrium-90 Resin Microspheres Versus Tyrosine Kinase Inhibitors in the Treatment of Unresectable Hepatocellular Carcinoma in France, Italy, Spain and the UK. J Med Econ.

[119] Krielen P, Grutters JP, Strik C, Ten Broek RP, Van Goor H, Stommel MW (2019). Cost-effectiveness of the Prevention of Adhesions and Adhesive Small Bowel Obstruction after Colorectal Surgery with Adhesion Barriers:A Modelling Study. World J Emerg Surg.

[120] Kolligs FT, Bilbao JI, Jakobs T, Iñarrairaegui M, Nagel JM, Rodriguez M (2015). Pilot Randomized Trial of Selective Internal Radiation Therapy vs. Chemoembolization in Unresectable Hepatocellular Carcinoma. Liver Int.

[121] Salem R, Gilbertsen M, Butt Z, Memon K, Vouche M, Hickey R (2013). Increased Quality of Life among Hepatocellular Carcinoma Patients Treated with Radioembolization, Compared with Chemoembolization. Clin Gastroenterol Hepatol.

[122] Barnacle AM, McHugh K (2006). Limitations with the Response Evaluation Criteria in Solid Tumors (RECIST) Guidance in Disseminated Pediatric Malignancy. Pediatr Blood Cancer.

[123] Mastrocostas K, Jang HJ, Fischer S, Dawson LA, Munoz-Schuffenegger P, Sapisochin G (2019). Imaging Post-stereotactic Body Radiation Therapy Responses for Hepatocellular Carcinoma:Typical Imaging Patterns and Pitfalls. Abdom Radiol.

[124] Hunter RD (1980). WHO Handbook for Reporting Results of Cancer Treatment. Int J Radiat Biol.

[125] Tovoli F, Renzulli M, Granito A, Golfieri R, Bolondi L (2017). Radiologic Criteria of Response to Systemic Treatments for Hepatocellular Carcinoma. Hepatic Oncol.

[126] Edeline J, Boucher E, Rolland Y, Vauléon E, Pracht M, Perrin C (2012). Comparison of Tumor Response by Response Evaluation Criteria in Solid Tumors (RECIST) and Modified RECIST in Patients Treated with Sorafenib for Hepatocellular Carcinoma. Cancer.

[127] Wald C, Russo MW, Heimbach JK, Hussain HK, Pomfret EA, Bruix J (2013). New OPTN/UNOS Policy for Liver Transplant Allocation:Standardization of Liver Imaging, Diagnosis, Classification, and Reporting of Hepatocellular Carcinoma. Radiology.

[128] Lee SH, Lee JM, Kim KW, Klotz E, Kim SH, Lee JY (2011). Dual-energy Computed Tomography to Assess Tumor Response to Hepatic Radiofrequency Ablation:Potential Diagnostic Value of Virtual Noncontrast Images and Iodine Maps. Invest Radiol.

[129] Gupta A, Gill A, Shrikanthan S, Srinivas S (2012). Nontargeted Y-90 Microsphere Radioembolization to Duodenum Visualized on Y-90 PET/CT and Bremsstrahlung SPECT/CT. Clin Nucl Med.

[130] Chiu RY, Yap WW, Patel R, Liu D, Klass D, Harris AC (2016). Hepatocellular Carcinoma Post Embolotherapy:Imaging Appearances and Pitfalls on Computed Tomography and Magnetic Resonance Imaging. Can Assoc Radiol J.

[131] Kim JW, Seong J, Yun M, Lee IJ, Yoon HI, Cho JH (2012). Usefulness of Positron Emission Tomography with Fluorine-18 Fluorodeoxyglucose in Predicting Treatment Response in Unresectable Hepatocellular Carcinoma Patients Treated with External Beam Radiotherapy. Int J Radiat Oncol Biol Phys.

[132] Takada Y, Kaido T, Shirabe K, Nagano H, Egawa H, Sugawara Y (2017). Significance of Preoperative Fluorodeoxyglucose-positron Emission Tomography in Prediction of Tumor Recurrence after Liver Transplantation for Hepatocellular Carcinoma Patients:A Japanese Multicenter Study. J Hepatobiliary Pancreat Sci.

[133] Hetta WM, Atyia HR (2020). Role of PET CT in Comparison to Triphasic CT in Early Follow-up of Hepatocellular Carcinoma after Transarterial Chemoemoblization. Egypt J Radiol Nucl Med.

[134] Song HJ, Cheng JY, Hu SL, Zhang GY, Fu Y, Zhang YJ (2015). Value of 18F-FDG PET/CT in Detecting Viable Tumour and Predicting Prognosis of Hepatocellular Carcinoma after TACE. Clin Radiol.

[135] Boas FE, Do B, Louie JD, Kothary N, Hwang GL, Kuo WT (2015). Optimal Imaging Surveillance Schedules after Liver-directed Therapy for Hepatocellular Carcinoma. J Vasc Interv Radiol.

[136] Lencioni R, Llovet JM (2010). Modified Recist (mRECIST) Assessment for Hepatocellular Carcinoma. Semin Liver Dis.

[137] Weng Z, Ertle J, Zheng S, Lauenstein T, Mueller S, Bockisch A (2013). Choi Criteria are Superior in Evaluating Tumor Response in Patients Treated with Transarterial Radioembolization for Hepatocellular Carcinoma. Oncol Lett.

[138] Schmidt N, Hess V, Zumbrunn T, Rothermundt C, Bongartz G, Potthast S (2013). Choi Response Criteria for Prediction of Survival in Patients with Metastatic Renal Cell Carcinoma Treated with Anti-angiogenic Therapies. Eur Radiol.

[139] Riaz A, Awais R, Salem R (2014). Side Effects of Yttrium-90 Radioembolization. Front Oncol.

[140] Kennedy AS, Coldwell D, Nutting C, Murthy R, Wertman DE, Loehr SP (2006). Resin 90Y-microsphere Brachytherapy for Unresectable Colorectal Liver Metastases:Modern USA Experience. Int J Radiat Oncol Biol Phys.

[141] Carr BI (2004). Hepatic Arterial 90Yttrium Glass Microspheres (therasphere) for Unresectable Hepatocellular Carcinoma:Interim Safety and Survival Data on 65 Patients. Liver Transplant.

[142] Jun BG, Kim YD, Cheon GJ, Kim ES, Jwa E, Kim SG (2018). Clinical Significance of Radiation-induced Liver Disease after Stereotactic Body Radiation Therapy for Hepatocellular Carcinoma. Korean J Intern Med.

[143] Hawkins MA, Dawson LA (2006). Radiation Therapy for Hepatocellular Carcinoma:From Palliation to Cure. Cancer.

[144] Hernando-Requejo O, Sánchez E, Fernández P, Zucca D, Pérez JM, García-Aranda M (2011). Institutional Experience on the Treatment of Lung and Liver Lesions with Stereotactic Body Radiotherapy and Novalis Exactrac Adaptive Gating Technique. J Radiosurgery SBRT.

[145] Gil-Alzugaray B, Chopitea A, Iñarrairaegui M, Bilbao JI, Rodriguez-Fraile M, Rodriguez J (2013). Prognostic Factors and Prevention of Radioembolization-induced Liver Disease. Hepatology.

[146] Braat MN, Van Erpecum KJ, Zonnenberg BA, Van Den Bosch MA, Lam MG (2017). Radioembolization-induced Liver Disease:A Systematic Review. Eur J Gastroenterol Hepatol.

[147] Guha C, Kavanagh BD (2011). Hepatic Radiation Toxicity:Avoidance and Amelioration. Semin Radiat Oncol.

[148] Oladeru OT, Miccio JA, Yang J, Xue Y, Ryu S, Stessin AM (2016). Conformal External Beam Radiation or Selective Internal Radiation Therapy-a Comparison of Treatment Outcomes for Hepatocellular Carcinoma. J Gastrointest Oncol.

[149] Wang TH, Huang PI, Hu YW, Lin KH, Liu CS, Lin YY (2018). Combined Yttrium-90 Microsphere Selective Internal Radiation Therapy and External Beam Radiotherapy in Patients with Hepatocellular Carcinoma:From Clinical Aspects to Dosimetry. PLoS One.

[150] Wu FX, Lu HR, Zhu SL, Li ZH, Zou L, Bai T (2016). Efficacy of Three-dimensional Conformal Radiotherapy Combined with Transarterial Chemoembolization for Hepatocellular Carcinoma with Portal Vein Tumor Thrombus. Onco Targets Ther.

[151] Kimura T, Aikata H, Doi Y, Imano N, Takeuchi Y, Takahashi I, Nishibuchi I (2018). Comparison of Stereotactic Body Radiation Therapy Combined with or without Transcatheter Arterial Chemoembolization for Patients with Small Hepatocellular Carcinoma Ineligible for Resection or Ablation Therapies. Technol Cancer Res Treat.

[152] Li XL, Guo WX, Hong XD, Yang L, Wang K (2016). Efficacy of the Treatment of Transarterial Chemoembolization Combined with Radiotherapy for Hepatocellular Carcinoma with Portal Vein Tumor Thrombus:A Propensity Score Analysis. Hepatol Res.

[153] Zhang T, Zhao YT, Wang Z, Li CR, Jin J, Jia AY (2016). Efficacy and Safety of Intensity-modulated Radiotherapy Following Transarterial Chemoembolization in Patients with Unresectable Hepatocellular Carcinoma. Med (United States).

[154] Bush DA, Smith JC, Slater JD, Volk ML, Reeves ME, Cheng J (2016). Randomized Clinical Trial Comparing Proton Beam Radiation Therapy with Transarterial Chemoembolization for Hepatocellular Carcinoma:Results of an Interim Analysis. Int J Radiat Oncol Biol Phys.

[155] Poon RT, Fan ST, Wong J (2000). Risk Factors, Prevention, and Management of Postoperative Recurrence After Resection of Hepatocellular Carcinoma. Ann Surg.

[156] Liu L, Shui Y, Yu Q, Guo Y, Zhang L, Zhou X (2021). Narrow-Margin Hepatectomy Resulted in Higher Recurrence and Lower Overall Survival for R0 Resection Hepatocellular Carcinoma. Front Oncol.

[157] Yu W, Wang W, Rong W, Wang L, Xu Q, Wu F (2014). Adjuvant Radiotherapy in Centrally Located Hepatocellular Carcinomas after Hepatectomy with Narrow Margin. J Am Coll Surg.

[158] Wei X, Jiang Y, Zhang X, Feng S, Zhou B, Ye X (2019). Neoadjuvant Three-dimensional Conformal Radiotherapy for Resectable Hepatocellular Carcinoma With Portal Vein Tumor Thrombus:A Randomized, Open-Label, Multicenter Controlled Study. J Clin Oncol.

[159] Lin H, Li X, Liu Y, Hu Y (2018). Neoadjuvant Radiotherapy Provided Survival Benefit Compared to Adjuvant Radiotherapy for Hepatocellular Carcinoma. ANZ J Surg.

[160] Mouiseddine M, François S, Souidi M, Chapel A (2012). Intravenous Human Mesenchymal Stem Cells Transplantation in NOD/SCID Mice Preserve Liver Integrity of Irradiation Damage. Methods Mol Biol.

[161] Chen YX, Zeng ZC, Sun J, Zeng HY, Yan-Huang Zhang ZY (2015). Mesenchymal Stem Cell-conditioned Medium Prevents Radiation-induced Liver Injury by Inhibiting Inflammation and Protecting Sinusoidal Endothelial Cells. J Radiat Res.

[162] Lee BM, Seong J (2021). Radiotherapy as an Immune Checkpoint Blockade Combination Strategy for Hepatocellular Carcinoma. World J Gastroenterol.

[163] Kim KJ, Kim JH, Lee SJ, Lee EJ, Shin EC, Seong J (2017). Radiation Improves Antitumor Effect of Immune Checkpoint Inhibitor in Murine Hepatocellular Carcinoma Model. Oncotarget.

[164] Chiang CL, Chan AC, Chiu KW, Kong FM (2019). Combined Stereotactic Body Radiotherapy and Checkpoint Inhibition in Unresectable Hepatocellular Carcinoma:A Potential Synergistic Treatment Strategy. Front Oncol.

[165] Tai WM, Loke KS, Gogna A, Tan SH, Ng DC, Hennedige TP (2020). A Phase II Open-label, Single-center, Nonrandomized Trial of Y90-radioembolization in Combination with Nivolumab in Asian Patients with Advanced Hepatocellular Carcinoma:CA 209-678. J Clin Oncol.

[166] Kudo M, Kawamura Y, Hasegawa K, Tateishi R, Kariyama K, Shiina S (2021). Management of Hepatocellular Carcinoma in Japan:JSH Consensus Statements and Recommendations 2021 Update. Liver Cancer.

[167] Korean Liver Cancer Association;National Cancer Center. 2018 Korean Liver Cancer Association-National Cancer Center Korea Practice Guidelines for the Management of Hepatocellular Carcinoma (2019). Gut Liver.

[168] Shiina S, Gani RA, Yokosuka O, Maruyama H, Nagamatsu H, Payawal DA (2020). APASL Practical Recommendations for the Management of Hepatocellular Carcinoma in the Era of COVID-19. Hepatol Int.

[169] Kumar A, Acharya SK, Singh SP, Arora A, Dhiman RK, Aggarwal R (2020). 2019 Update of Indian National Association for Study of the Liver Consensus on Prevention, Diagnosis, and Management of Hepatocellular Carcinoma in India:The Puri II Recommendations. J Clin Exp Hepatol.

[170] Heimbach JK, Kulik LM, Finn RS, Sirlin CB, Abecassis MM, Roberts LR (2018). AASLD Guidelines for the Treatment of Hepatocellular Carcinoma. Hepatology.

[171] Piñero F, Tanno M, Soteras GA, Baña MT, Dirchwolf M, Fassio E (2020). Argentinian Clinical Practice Guideline for Surveillance, Diagnosis, Staging and Treatment of Hepatocellular Carcinoma. Ann Hepatol.

[172] Chagas AL, de Mattos AA, Carrilho FJ, Bittencourt PL, Vezozzo DC, Horvat N (2020). Brazilian Society of Hepatology Updated Recommendations for Diagnosis and Treatment of Hepatocellular Carcinoma. Arq Gastroenterol.

[173] European Association for the Study of the Liver. Electronic address:easloffice@easloffice.eu;European Association for the Study of the Liver. EASL Clinical Practice Guidelines:Management of Hepatocellular Carcinoma (2018). J Hepatol.

[174] Benson AB, Abbott DE, Anaya DA, Anders R, Bachini M, Burgoyne A (2021). NCCN Guidelines Version 3 2021. Hepatobiliary Cancers.

[175] Vogel A, Cervantes A, Chau I, Daniele B, Llovet JM, Meyer T (2018). Hepatocellular Carcinoma:ESMO Clinical Practice Guidelines for Diagnosis, Treatment and Follow-up. Ann Oncol.

[176] Shao YY, Wang SY, Lin SM (2021). Diagnosis Group;Systemic Therapy Group. Management Consensus Guideline for Hepatocellular Carcinoma:2020 Update on Surveillance, Diagnosis, and Systemic Treatment by the Taiwan Liver Cancer Association and the Gastroenterological Society of Taiwan. J Formos Med Assoc.

[177] Meyers BM, Knox J, Cosby R, Beecroft JR, Chan KK, Coburn N (2020). Nonsurgical Management of Advanced Hepatocellular Carcinoma:A Clinical Practice Guideline. Curr Oncol.

[178] Chen LT, Martinelli E, Cheng AL, Pentheroudakis G, Qin S, Bhattacharyya GS (2020). Pan-Asian Adapted ESMO Clinical Practice Guidelines for the Management of Patients with Intermediate and Advanced/Relapsed Hepatocellular Carcinoma:A TOS-ESMO Initiative Endorsed by CSCO, ISMPO, JSMO, KSMO, MOS and SSO. Ann Oncol.

[179] Alqahtani S, Sanai F, Alolayan A, Abaalkhail F, Alsuhaibani H, Hassanain M (2020). Saudi Association for the Study of Liver Diseases and Transplantation Practice Guidelines on the Diagnosis and Management of Hepatocellular Carcinoma. Saudi J Gastroenterol.

