# Erratum

**Published:** 2021-12-10

*From the editor*

The statement about Creative Commons license in all published articles of Volumes 1–6 was incomplete and ambiguous. For the purpose of clarity and completeness, we would like to amend this statement to as follows: This is an Open Access article distributed under the terms of the Creative Commons Attribution - Non Commercial 4.0 International License (CC BY-NC 4.0), permitting all non-commercial use, distribution, and reproduction in any medium, provided the original work is properly cited.

Moreover, we hereby apologize for the omission of conflict of interest disclosures on certain articles published in Volumes 1, 2, 3, 5, and 6, including several Special Issues, of *Journal of Clinical and Translational Research*. See below for the conflict of interest statements of the affected articles.

***Correction***

Dirkes MC, van Gulik TM, Heger M. Survey and critical appraisal of pharmacological agents with potential thermo-modulatory properties in the context of artificially induced hypometabolism. J Clin Transl Res 2015;1:6-21. DOI: 10.18053/jctres.201501.003

The conflict of interest statement was unintentionally omitted due to technical issue.

After confirming with the corresponding author again, the conflict of interest statement should have been as follows: “No conflict of interest was reported by the authors.”

***Correction***

Heger M. Editor’s inaugural issue foreword: perspectives on translational and clinical research. J Clin Transl Res 2015;1:1-5. DOI: 10.18053/jctres.201501.005

The conflict of interest statement was unintentionally omitted due to technical issue.

After confirming with the corresponding author again, the conflict of interest statement should have been as follows: “No conflict of interest was reported by the author.”

***Correction***

Pabittei DR, Heger M, Simonet M, van Tuijl S, van der Wal AC, van Bavel E, *et al*. Laser-assisted vascular welding: optimization of acute and post-hydration welding strength. J Clin Transl Res 2015;1:31-47. DOI: 10.18053/jctres.201501.001

The conflict of interest statement was unintentionally omitted due to technical issues.

After confirming with the corresponding author again, the conflict of interest statement should have been as follows: “No conflict of interest was reported by the authors.”

***Correction***

Dirkes MC, van Gulik TM, Heger M. The physiology of artificial hibernation. J Clin Transl Res 2015;1:78-93. DOI: 10.18053/jctres.201502.005

The conflict of interest statement was unintentionally omitted due to technical issue.

After confirming with the corresponding author again, the conflict of interest statement should have been as follows: “No conflict of interest was reported by the authors.”

***Correction***

Pabittei DR, de Boon W, Heger M, van Golen RF, Balm R, Legemate DA, *et al*. Laser-assisted vessel welding: state of the art and future outlook. J Clin Transl Res 2015;1:61-77. DOI: 10.18053/jctres.201502.006

The conflict of interest statement was unintentionally omitted due to technical issue.

After confirming with the corresponding author again, the conflict of interest statement should have been as follows: “No conflict of interest was reported by the authors.”

***Correction***

van Golen RF, Stevens KM, Colarusso P, Jaeschke H, Heger M. Platelet aggregation but not activation and degranulation during the acute post-ischemic reperfusion phase in livers with no underlying disease. J Clin Transl Res 2015;1:107-115. DOI: 10.18053/jctres.201502.001

The conflict of interest statement was unintentionally omitted due to technical issue.

After confirming with the corresponding author again, the conflict of interest statement should have been as follows: “No conflict of interest was reported by the authors.”

***Correction***

Liu Y, Qin R, Zaat SAJ, Breukink E, Heger M. Antibacterial photodynamic therapy: overview of a promising approach to fight antibiotic-resistant bacterial infections. J Clin Transl Res 2015;1:140-167. DOI: 10.18053/jctres.201503.002

The conflict of interest statement was unintentionally omitted due to technical issue.

After confirming with the corresponding author again, the conflict of interest statement should have been as follows: “No conflict of interest was reported by the authors.”

***Correction***

Lopez J, Richardson E, Tiozzo E, Lantigua L, Martinez C, Abreut G, *et al*. The effect of exercise training on disease progression, fitness, quality of life, and mental health in people living with HIV on antiretroviral therapy: a systematic review. J Clin Transl Res 2015;1:129-139. DOI: 10.18053/jctres.201503.001

The conflict of interest statement was unintentionally omitted due to technical issue.

After confirming with the corresponding author again, the conflict of interest statement should have been as follows: “The authors declare no conflicts of interest.”

***Correction***

Quintin L, Karemaker JM, McAllen R. Cardiac vagal activity and daily clinical practice. J Clin Transl Res 2016;2:1-2. DOI: 10.18053/jctres.02.201601.002

The conflict of interest statement was unintentionally omitted due to technical issue.

After confirming with the corresponding author again, the conflict of interest statement should have been as follows: “No conflict of interest was reported by the authors.”

***Correction***

Smith AP. Chewing gum and stress reduction. J Clin Transl Res 2016;2:52-54. DOI: 10.18053/jctres.02.201602.002

The conflict of interest statement was unintentionally omitted due to technical issue.

After confirming with the corresponding author again, the conflict of interest statement should have been as follows: “No conflict of interest was reported by the author.”

***Correction***

Martin A, Stillman J, Miguez M-J, McDaniel HR, Konefal J, Woolger JM, *et al*. The effect of dietary supplementation on brain-derived neurotrophic factor and cognitive functioning in Alzheimer’s dementia. J Clin Transl Res 2017;3:337-343. DOI: 10.18053/jctres.03.201703.006

The conflict of interest statement was unintentionally omitted due to technical issue.

After confirming with the corresponding author again, the conflict of interest statement should have been as follows: “H. Reginald McDaniel has received income as a manufacturer of the APMC dietary supplement. Jordan Stillman, Alicia Martin, Maria-Jose Miguez, Janet Konefal, Judi M. Woolger, and John E. Lewis have no conflicts of interest to report.”

***Correction***

Boyle G. Proving a negative? Methodological, statistical, and psychometric flaws in Ullmann *et al*. (2017) PTSD study. J Clin Transl Res 2017;3:375-381. DOI: 10.18053/jctres.03.2017S2.005 The conflict of interest statement was unintentionally omitted due to technical issue.

After confirming with the corresponding author again, the conflict of interest statement should have been as follows: “No conflict of interest was reported by the author.”

***Correction***

Earp BD. The need for reporting negative results - a 90 year update. J Clin Transl Res 2017;3:1-4. DOI: 10.18053/jctres.03.2017S2.001

The conflict of interest statement was unintentionally omitted due to technical issue.

After confirming with the corresponding author again, the conflict of interest statement should have been as follows: “No conflict of interest was reported by the author.”

***Correction***

Earp BD, Wilkinson D. The publication symmetry test: a simple editorial heuristic to combat publication bias. J Clin Transl Res 2017;3:5-7. DOI: 10.18053/jctres.03.2017S2.002

The conflict of interest statement was unintentionally omitted due to technical issue.

After confirming with the corresponding author again, the conflict of interest statement should have been as follows: “No conflict of interest was reported by the authors.”

***Correction***

Mesko B. Health IT and digital health: the future of health technology is diverse. J Clin Transl Res 2017;3:431-434. DOI: 10.18053/jctres.03.2017S3.006

The conflict of interest statement was unintentionally omitted due to technical issue.

After confirming with the corresponding author again, the conflict of interest statement should have been as follows: “No conflict of interest was reported by the author.”

***Correction***

Teoh N, Farrell G. Statins as early therapy to mitigate COVID-19 (SARS-CoV-2)-associated ARDS and cytokine storm syndrome – time is of the essence. J Clin Transl Res 2020;5:227-229. DOI: 10.18053/jctres.05.202005.001

The conflict of interest statement was unintentionally omitted due to technical issue.

After confirming with the corresponding author again, the conflict of interest statement should have been as follows: “No conflict of interest was reported by the authors.”

***Correction***

Sakthivel S, Gayathri V, Anirudhan S, Jaya Shree Roja R. Platelet-rich fibrin and collagen matrix for the regeneration of infected necrotic immature teeth. J Clin Transl Res 2020;6:1-5. DOI: 10.18053/jctres.06.202001.001

The conflict of interest statement was unintentionally omitted due to technical issue.

After confirming with the corresponding author again, the conflict of interest statement should have been as follows: “No conflict of interest was reported by the authors.”

***Correction***

Qasim A, Kousa O, Andukuri VG. Hypolipidemia contributing to the severity of sepsis triggered by influenza A virus: a case report. J Clin Transl Res 2020;6:198-202. DOI: 10.18053/jctres.06.202006.002

The conflict of interest statement was unintentionally omitted due to technical issue.

After confirming with the corresponding author again, the conflict of interest statement should have been as follows: “No conflict of interest was reported by the authors.”

